# The statistical importance of a study for a network meta-analysis estimate

**DOI:** 10.1186/s12874-020-01075-y

**Published:** 2020-07-16

**Authors:** Gerta Rücker, Adriani Nikolakopoulou, Theodoros Papakonstantinou, Georgia Salanti, Richard D. Riley, Guido Schwarzer

**Affiliations:** 1 Institute of Medical Biometry and Statistics, Faculty of Medicine and Medical Center - University of Freiburg, Stefan-Meier-Strasse 26, Freiburg, 79104 Germany; 2 Institute of Social and Preventive Medicine (ISPM), University of Bern, Bern, Switzerland; 3 Centre for Prognosis Research, Research Institute for Primary Care and Health Sciences, Keele University, Keele, UK

**Keywords:** Network meta-analysis, Study weight, Study contribution, Study importance

## Abstract

**Background:**

In pairwise meta-analysis, the contribution of each study to the pooled estimate is given by its weight, which is based on the inverse variance of the estimate from that study. For network meta-analysis (NMA), the contribution of direct (and indirect) evidence is easily obtained from the diagonal elements of a hat matrix. It is, however, not fully clear how to generalize this to the percentage contribution of each study to a NMA estimate.

**Methods:**

We define the importance of each study for a NMA estimate by the reduction of the estimate’s variance when adding the given study to the others. An equivalent interpretation is the relative loss in precision when the study is left out. Importances are values between 0 and 1. An importance of 1 means that the study is an essential link of the pathway in the network connecting one of the treatments with another.

**Results:**

Importances can be defined for two-stage and one-stage NMA. These numbers in general do not add to one and thus cannot be interpreted as ‘percentage contributions’. After briefly discussing other available approaches, we question whether it is possible to obtain unique percentage contributions for NMA.

**Conclusions:**

Importances generalize the concept of weights in pairwise meta-analysis in a natural way. Moreover, they are uniquely defined, easily calculated, and have an intuitive interpretation. We give some real examples for illustration.

## Background

Pairwise meta-analysis (pairwise MA) is used to summarize the evidence for a treatment effect from all eligible studies that compared the two interventions of interest. In two-stage pairwise MA, the contribution of each study to the pooled estimate is measured by its weight, which depends on the type of data, the chosen summary measure, and the chosen statistical model. For example, for mean differences usually the inverse of the variance of the estimated mean difference for a study is used as that study’s weight, though this is an estimated, not a fixed number.

Network meta-analysis (NMA) extends the pairwise MA approach to an arbitrary number of interventions. It is usually based on a set of randomized trials, each comparing a subset of two or more of the treatments that are of interest for the underlying research question. The evidence from these studies is then put together in a model, preferably respecting the principle of concurrent control by using a model that is based on the within-study treatment contrasts [1].

The objective is to describe how important each study is for the estimate of a given treatment effect in the NMA. We distinguish between approaches that focus on the study’s *contribution* in the sense of attributing a ‘weight’ to each study, mainly depending on the variances, and approaches that also take into account the direction and size of each treatment effect, and which are seeking for ‘influential’ or explicitly ‘outlying’ studies. For the latter, see [2–5]. In this paper, we concentrate on the first approach; that is, we are mainly interested in measuring study contributions without looking at their effect estimates.

For NMA, while several methods exist to obtain the contribution of direct (and indirect) evidence of each comparison to its own NMA estimate, it is far less obvious how to define the contribution, or the importance, of each study to any (other) treatment effect estimate. Several proposals exist in the literature, based on different approaches. Most of them come with some limitations; their results also do not in general agree [6–9].

The proportions of direct and indirect evidence have been investigated in the past. The method of ‘back-calculation’, which we describe in the “Methods” section, goes back to Bucher’s work [10] and was proposed by Dias and others in a Bayesian framework [11]. It was also given in a frequentist context [12]. In NMA based on the inverse variance method, NMA estimates are linear combinations of treatment effect estimates from primary studies with coefficients that constitute the rows of the hat matrix. The direct evidence proportion of a study or a comparison is easily obtained from the diagonal elements of the respective hat matrix [12]. As an alternative, Dias and others suggested ‘node splitting’, which means estimating the indirect evidence for a comparison by modeling out all studies that provide direct information for this comparison [11]. This method was developed further [13] and called ‘side splitting’ by others [14]. Whereas White [14] interpreted the term ‘side’ as an edge in the network graph, others used SIDE as an abbreviation of ‘Separating Indirect and Direct Evidence’ [15, 16]. Noma and others proposed quantifying the indirect evidence based on a factorization of the total likelihood into separate component likelihoods [17]. So far, these authors did not undertake to define or estimate the contribution of each study to a given comparison in the network.

A proposal to this aim, based on the off-diagonal elements of the hat matrix, was made by Salanti and others [6]. Implicitly, this idea also underlies the net heat plot that was suggested by Krahn and others, a heatmap visualization representing the absolute size of the hat matrix elements by gray squares [18]. However, as an approach to define percentage study contributions, it has problems, as the hat matrix elements are signed and do not add to 1. Papakonstantinou and others, acknowledging this limitation, developed a different concept, likewise based on the hat matrix, using ideas by König and others [12], and successfully avoided these deficiencies [9]. Although the proposed algorithm is not strictly deterministic, it was empirically shown that this did not materially affect the estimated percentage contributions. These contributions are currently used in the software CINeMA (Confidence in Network Meta-Analysis) to investigate the trustworthiness of each comparison’s NMA estimate, based on the risk of bias (and other features) of the individual studies that contribute to the comparison [9, 19].

Another approach to percentage contributions was published by Jackson and others [7] and also used elsewhere [8, 20]. They suggest decomposing the total variance matrix of parameter estimates from the meta-analysis via a decomposition of the observed Fisher information matrix into independent study-specific contributions, which sum up to the total variance matrix. Their diagonal elements can be used to derive percentage study weights for each parameter. They reveal how the variance of a parameter is changed by the inclusion of a particular study, assuming that all variance estimates (within- and between-study variances) are fixed at the same value as in the full analysis of all studies. Although this approach is adequate when within-study information is pooled across studies, the study-specific contributions can become distorted in situations where across-study information contributes to the parameter estimates, as in a network meta-analysis [9].

In the present paper we suggest an approach that does not require that the contributions to each network estimate sum to 100%. The structure of the article is as follows. In the “Methods” we introduce our notation and give the definition of the statistical importance of a study to a network meta-analysis estimate. We then show that this is a generalization of both the weights in a pairwise meta-analysis and the direct and indirect evidence proportions in a NMA. We give several interpretations of the quantity ‘direct evidence proportion’ and show that starting from different interpretations of this quantity leads to different generalizations. In the next section we present two real data sets for illustrating our method. In the “Results” we illustrate our concept by first applying it to simple special cases and standard networks like pairwise MA, a chain of treatments, and a circle, and then apply it to the two real data examples. We discuss strengths and limitations of our approach in the “Discussion”, and the paper ends with a recommendation in the “Conclusion” sections.

## Methods

We start with defining the importance of a study for any network comparison in the framework of the common effect model (traditionally termed ‘fixed effect model’). We then show how our measure of importance is related to the proportions of direct and indirect evidence for a NMA estimate and give various interpretations for that. We also extend it to the random effects model. While the common effect model assumes that, for each comparison, all studies in the network are estimating the same (comparison-specific) true effect, the random effects model assumes that the underlying effects of each comparison follow a distribution. Often a normal distribution is assumed [21].

### The importance of each study: variance reduction by adding direct information

Consider a network meta-analysis. First concentrating on the common effect model, we ask for the amount by which the variance of an estimate from only indirect evidence is reduced if direct information is added, or the relative loss in precision when direct evidence is removed. There is no reason to assume that these quantities add up to 100%. We will come back to this point later.

The *importance of each study* for each NMA estimate is defined as follows:

Conduct a NMA for the given network, called NMA_*all*_, and then repeat the following steps for each study *i* in turn:

1. Remove study *i* from the network.
2. Conduct a NMA for the network without study *i*. Let us denote this result by NMA_−*i*_. Accordingly, denote the variance of any treatment effect estimate *c* by *V*_*all*_(*c*) if estimation is based on NMA_*all*_ and by *V*_−*i*_(*c*) if estimation is based on NMA_−*i*_.
3. For all comparisons *c*, define the importance of study *i* for comparison *c* as
  1 $$  p(i,c) = 1 - \frac{V_{all}(c)}{V_{-i}(c)} = \frac{V_{-i}(c) - V_{all}(c)}{V_{-i}(c)},  $$
  thus giving the reduction of the variance of comparison *c* with respect to the reduced network, if the removed study is reinstalled.

The first step (removing study *i* from the network) could lead to a disconnected network, rendering the calculation of *V*_−*i*_(*c*) impossible. For implementation in practice, instead of removing study *i*, we set the standard errors for all comparisons from study *i* to a very large value(e.g., 10000), thus downweighting study *i* to practically zero. This approach, known as ‘data augmentation’, goes back to White and others [22] and was also used by Riley and others [8]. While the interpretation of the difference of the variances (numerator of (1)) depends on the particular scale, the proposed measure is dimensionless. We have 0≤*p*(*i*,*c*)≤1 for all studies *i* and comparisons *c*. We emphasize, however, that it makes no sense to add up these importances across all studies, as they do not sum up to 100% (in fact, the sum is often larger). We do not call them (percentage) contributions. Rather, they measure the importance of a study for a comparison.

The idea can be illustrated by comparing a network of studies to a network of roads in a town. We consider the traffic from some place A (a node in the network) to another place B. The precision (or the weight) can be interpreted as the transport capacity of the road network between A and B, comparable to the conductance in an electrical network [23]. If a particular road is closed due to construction works, a traffic accident, or flooding, many capacities decrease because some people have to make a detour to go from A to B and thus add to the traffic on other roads. The *importance* of this road for the way from A to B is given by the relative reduction of the capacity of the network due to the road closure.

The algorithm to calculate importances is implemented in R function netimpact() in the R package **netmeta** [24, 25], with a data set about Parkinson’s disease as example. R code for all examples can be found in Additional File 1, with the resulting plots shown in Additional File 2.

### The importance of a comparison for itself: direct and indirect evidence proportions

In this paragraph, we show how our definition of importance was motivated by (but is not limited to) the known concepts of direct and indirect evidence proportions in the context of two-stage meta-analysis with inverse variance weights, still based on the common effect model.

We denote the variance estimates of the NMA effect estimate, the direct effect estimate and the indirect effect estimate of a comparison *c* by *V*_*nma*_(*c*),*V*_*dir*_(*c*),*V*_*ind*_(*c*) and the inverse variance weights by $w_{nma}(c) = \left [V_{nma}(c)\right ]^{-1}, \dots $ and so on. These weights are quantities that must be estimated from the data. Direct and indirect paths and thus effects can be assumed as independent. For readability, we omit hats on the symbols. The rules of variance calculation lead to

2 $$  V_{nma}(c) = \left(\frac{1}{V_{dir}(c)} + \frac{1}{V_{ind}(c)}\right)^{-1} = \frac{V_{dir}(c) \cdot V_{ind}(c)}{V_{dir}(c) + V_{ind}(c)}  $$

[10, 11] and we may write

3 $$ \frac{V_{nma}(c)}{V_{ind}(c)} = \frac{V_{dir}(c) - V_{nma}(c)}{V_{dir}(c)} = 1 - \frac{V_{nma}(c)}{V_{dir}(c)}  $$

4 $$ \frac{V_{nma}(c)}{V_{dir}(c)} = 1 - \frac{V_{nma}(c)}{V_{ind}(c)} = \frac{V_{ind}(c) - V_{nma}(c)}{V_{ind}(c)}  $$

and in terms of inverse variance weights

5 $$  \frac{V_{nma}(c)}{V_{dir}(c)} = 1 - \frac{w_{ind}(c)}{w_{nma}(c)} = \frac{w_{nma}(c) - w_{ind}(c)}{w_{nma}(c)}.  $$

The direct evidence proportion of comparison *c* can be defined via the inverse variance weights as

6 $$  p(c) := \frac{w_{dir}(c)}{w_{dir}(c) + w_{ind}(c)} = \frac{V_{ind}(c)}{V_{dir}(c) + V_{ind}(c)}.  $$

Inserting (2) into (6) and using also (3) and (4), we obtain for the proportions of direct and indirect evidence

7 $$  p(c) = \frac{V_{nma}(c)}{V_{dir}(c)} ; \quad 1 - p(c) = \frac{V_{nma}(c)}{V_{ind}(c)}.  $$

In practice, users of the R package **netmeta** obtain the values of *p*(*c*) via function netmeasures() [24]. We now show that different interpretations for *p*(*c*) are possible and that these lead to different concepts of generalization.

#### Interpretation 1: proportion of direct evidence

The first interpretation of *p*(*c*), an immediate consequence of the definition (6), is that it describes the proportion of network precision for comparison *c* attributed to direct evidence (from pairwise MA), in short, the contribution of direct evidence to the network estimate *c*. Accordingly, 1−*p*(*c*) represents the contribution of indirect evidence to this estimate.

#### Interpretation 2: reduction of the variance of a direct comparison by adding indirect information

Equation (7) provides another interpretation: *p*(*c*) is the proportion to which the variance of a pairwise MA is shrunk when indirect evidence from the whole network is added to the direct evidence, or, in other words, when all network information is used.

#### Interpretation 3: relative reduction of the variance of a comparison based solely on indirect evidence when adding direct information

This interpretation is suggested by equation (4): *p*(*c*) is the relative reduction of the variance of a comparison with only indirect evidence when information from the direct comparison is added. Similarly, *p*(*c*) could be interpreted as the loss in precision of a comparison when removing direct evidence (i.e., using exclusively indirect evidence). This interpretation is motivated by equation (5).

Whatever interpretation is preferred, high values mean a high importance of the direct comparison for itself, and low values mean low importance. The concept of ‘direct evidence proportion’ quantifies the contribution of the direct evidence from a comparison to *its own* NMA estimate. Our definition (1) generalizes this to the importance of a comparison/a study for the NMA estimate of *any (other)* comparison.

### Extension to the random effects model

So far, we used the common effect model to derive the importances. The estimate $\hat \tau ^{2}$ of the variance of the random effects did not enter the calculations. Leaving out a study from the data is expected to change $\hat \tau ^{2}$. Particularly, the variance may *decrease* if the omitted study contributed a lot to the between-study heterogeneity or inconsistency, resulting in a negative importance for this comparison. A possible workaround is to insert the estimate $\hat \tau ^{2}$ from the original network as a common heterogeneity variance estimate for all subnetworks [7]. For pairwise MA, this leads to the usual random effects weights, as we will see in the next subsection.

### Pairwise meta-analysis

We consider a pairwise MA with inverse variance weighting, such that the (unstandardized) weight of study *i* is given by *w*_*i*_=1/*V*_*i*_ where *V*_*i*_ is the (estimated) variance of study *i*. The variance of the pooled common effect estimate is then estimated by $1/{\sum \nolimits }_{j} w_{j}$. Removing study *i* from the MA gives another pooled estimate with variance $1/{\sum \nolimits }_{j \ne i} w_{j}$. Equation (5) provides the importance of study *i* for the pooled estimate

$$\frac{{\sum\nolimits}_{j} w_{j} - {\sum\nolimits}_{j \ne i} w_{j}}{{\sum\nolimits}_{j} w_{j}} = \frac{w_{i}}{{\sum\nolimits}_{j} w_{j}}$$

which is the relative weight of study *i* in line with what we would expect. For the random effects model, we use an estimate of the heterogeneity variance $\hat \tau ^{2}$ for the full pairwise MA, and then remove one study in turn while fixing the heterogeneity variance to this value. The same argumentation as above shows that the procedure leads to the usual random effects weights, $w_{i}^{*} = 1/\left (V_{i} + \hat \tau ^{2}\right)$ and corresponding relative weights.

This equality of weights is exact only if inverse variance weighting is used (e.g., for mean differences or Peto odds ratios) and strictly only if these variances are known (which is not true in practice), but not in general. For example, due to the different weighting method, it does not hold exactly for binary outcomes when using the Mantel-Haenszel method.

### One-stage network meta-analysis

Of note, however, the importance concept allows approximate ‘study weights’ to be derived also for one-stage pairwise meta-analyses based on a generalized linear model, such as logistic regression, where study weights are not commonly provided. Importances can also be derived from a one-stage approach based on the Mantel-Haenszel method for NMA [26]. We provide an example in Additional File 1.

This allows deriving not only study weights, but also direct and indirect evidence proportions for one-stage NMA. For a comparison *c*, consider the network meta-analysis of all studies except those that include *c*, and let *V*_−*c*_(*c*) be the variance of the NMA effect estimate of *c* for this reduced network. In analogy to (1), we may define the direct evidence proportion of comparison *c* as

$$\begin{array}{@{}rcl@{}} p(c,c) = 1 - \frac{V_{all}(c)}{V_{-c}(c)} = \frac{V_{-c}(c) - V_{all}(c)}{V_{-c}(c)} \end{array} $$

and the indirect evidence proportion as 1−*p*(*c*,*c*)=*V*_*all*_(*c*)/*V*_−*c*_(*c*). We suggest also removing multi-arm studies that include *c*, following the ‘separate indirect from direct design evidence’ (SIDDE) approach suggested by Efthimiou et al. [26].

### Data sets

#### Parkinson’s data

This network consists of seven studies comparing five treatments: placebo, coded 1, and four active drugs, pramipexole (coded 2), ropinirole (3), bromocriptine (4), and cabergoline (5) [27]. The outcome is the mean lost work-time reduction in patients given dopamine agonists as adjunct therapy in Parkinson’s disease, given as sample size, mean and standard deviation in each trial arm. The data, shown in Table 1, is used as an example in the supplementary material of [28] and available from the R package **netmeta** [24], see the R code in Additional file 1.

**Table 1:** Parkinson’s data. mean = mean lost worktime reduction, sd = standard deviation, n = sample size

		Arm 1				Arm 2				Arm 3		
Study	Treatment	mean	sd	n	Treatment	mean	sd	n	Treatment	mean	sd	n
1	placebo	-1.22	3.70	54	ropinirole	-1.53	4.28	95				
2	placebo	-0.70	3.70	172	pramipexole	-2.40	3.40	173				
3	placebo	-0.30	4.40	76	pramipexole	-2.60	4.30	71	bromocriptine	-1.2	4.3	81
4	ropinirole	-0.24	3.00	128	bromocriptine	-0.59	3.00	72				
5	ropinirole	-0.73	3.00	80	bromocriptine	-0.18	3.00	46				
6	bromocriptine	-2.20	2.31	137	cabergoline	-2.50	2.18	131				
7	bromocriptine	-1.80	2.48	154	cabergoline	-2.10	2.99	143				

#### Thrombolytic data

**Fig. 1:**
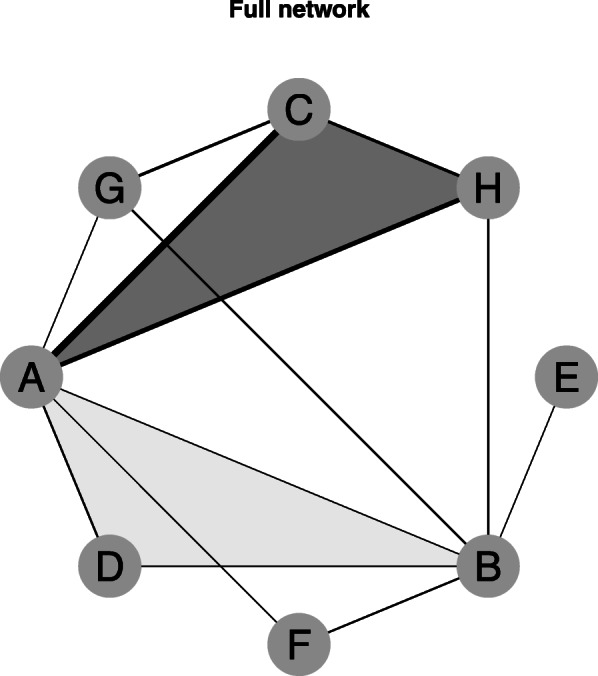
Network graph of thrombolytic data. The gray shaded areas indicate two three-arm studies: study 1 (lightgray, A-B-D), and study 2 (darkgray, A-C-H). A = streptokinase, B = accelerated alteplase, C = alteplase, D = streptokinase + alteplase, E = tenecteplase, F = reteplase, G = urokinase, H = anistreptilase

This data set, originally published by Boland and others [29], was extended and presented by Lu and Ades [30] and successively analyzed by many others. We took the data from Riley and others [8]. The outcome is mortality at 30-35 days. This network consists of 28 studies (13 designs, i.e., different combinations of treatments in a study) of 8 treatments after acute myocardial infarction. We follow Riley and others [8] denoting these treatments by A = streptokinase, B = accelerated alteplase, C = alteplase, D = streptokinase + alteplase, E = tenecteplase, F = reteplase, G = urokinase, and H = anistreptilase. Figure 1 shows the network graph for the thrombolytic data which are provided in Additional File 3.

## Results

We first apply our method to a number of hypothetical examples that nevertheless lead to insight into the interpretation of our new measure of importance.

### Hypothetical networks

#### A chain of *n*−1 studies connecting *n* treatments

Suppose we have three studies comparing A to B, B to C, and C to D with variances *V*_1_,*V*_2_,*V*_3_. We look at comparison A:D. The direct evidence proportion for comparison A:D is 0, the indirect evidence proportion is 1. The variance of the NMA (i.e., the indirect) estimate for comparison A:D is *V*_*all*_(A:D)=*V*_1_+*V*_2_+*V*_3_. If one of the studies, regardless which, is omitted, the variance becomes infinite, and the importance of this study for comparison A:D becomes 1. The interpretation is that this study (like the others) is of maximum importance for the comparison, which is indeed true. For the approach by Papakonstantinou and others, henceforth called contributions approach, each study would contribute 1/3 to the estimate of comparison A:D [9].

#### A circle of *n* treatments with equal variances

**Fig. 2:**
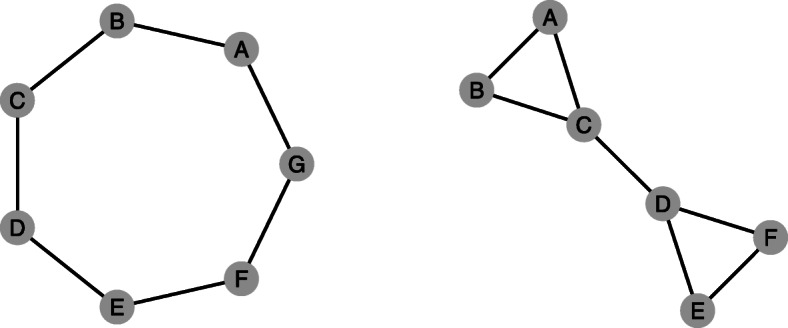
Left panel: A circle of *n* treatments with equal variances. Right panel: A network with a bridge

Suppose we have a closed circular network of *n* treatments connected by exactly *n* two-arm studies, each comparing two treatments in turn like in Fig. 2 (left, here *n*=7) and each having variance 1.

We consider an arbitrary comparison *c*=*t*_1_:*t*_2_ of treatments *t*_1_ and *t*_2_ (*t*_1_≠*t*_2_) in the network such that the distance (the number of steps) from *t*_1_ to *t*_2_ is 1≤*l*≤*n*/2. The NMA variance of comparison *c* is

$$V_{all}(c) = \frac{1}{\frac{1}{l} + \frac{1}{n-l}} = \frac{l(n-l)}{n}.$$

For example, comparison A:D (distance 3) has the NMA variance 3*4/7. If a study is removed from the network, evidence for comparison *c* can only go one way. If, for example, the study connecting B and C is removed from the network, the variance for comparison A:D becomes 4, because the shorter connection via B and C is broken. In general, if the removed study lies on the shorter path from *t*_1_ to *t*_2_ (length *l*), the variance becomes *n*−*l*, if the removed study lies on the longer path, the variance becomes *l*. Thus the importance of each study *i* on the shorter path for comparison *c* is

$$p(i,c) = 1 - \frac{V_{all}(c)} {V_{-i}(c)} = 1 - \frac{l(n-l)}{n(n-l)} = 1 - \frac{l}{n} = \frac{n-l}{n} $$

and the importance of each study *j* on the longer path for comparison *c* is

$$p(j,c) = 1 - \frac{V_{all}(c)} {V_{-j}(c)} = 1 - \frac{l(n-l)}{nl} = 1 - \frac{n-l}{n} = \frac{l}{n}.$$

Thus, plausibly, each of the *l* studies on the shorter path has greater importance for the comparison than the *n*−*l* studies on the longer path, as we have presumed *l*≤*n*−*l*. For the example, the importance of study B:C for comparison A:D is (7 - 3)/7 = 4/7. Particularly, it follows that the direct evidence proportion for each pair that is directly compared (i.e., adjacent, *l*=1) is (*n*−1)/*n* and the indirect evidence proportion is 1/*n*, while for all other pairs the direct evidence proportion is 0 and the indirect evidence proportion is 1. By contrast, the contributions approach would attribute a contribution of $\frac {n-l}{nl}$ to each piece on the shorter path and $\frac {l}{n(n-l)}$ to each piece on the longer path, such that the sum of all contributions is 1.

#### A network with a bridge

We consider the network given on the right-hand panel of Fig. 2 with seven studies, all again having variance 1. We call comparison C:D a bridge [9, Supplementary file 3]. Table 2 gives the importances of each study for all comparisons. Study CD has importance 1 for all comparisons between the two parts of the network (A:D, A:E, A:F, B:D, B:E, B:F, C:D, C:E, C:F) and importance 0 for all comparisons within the same part of the network (A:B, A:C, B:C, D:E, D:F, E:F). Studies AB, AC and BC have no importance for comparisons outside the triangle ABC (C:D, C:E, C:F, D:E, D:F, E:F), and vice versa for triangle DEF. The table also shows that the direct evidence proportion for comparisons A:B, A:C, B:C, D:E, D:F, and E:F is 2/3, and the direct evidence proportion for comparison C:D (the bridge) is 1.

**Table 2:** Importances of each study for the network with a bridge. For sake of transparency, the symbol − represents zero

Comparisons															
Study	A:B	A:C	B:C	C:D	D:E	D:F	E:F	A:D	B:D	C:E	C:F	A:E	A:F	B:E	B:F
AB	0.67	0.33	0.33	–	–	–	–	0.17	0.17	–	–	0.12	0.12	0.12	0.12
AC	0.33	0.67	0.33	–	–	–	–	0.44	0.17	–	–	0.36	0.36	0.12	0.12
BC	0.33	0.33	0.67	–	–	–	–	0.17	0.44	–	–	0.12	0.12	0.36	0.36
CD	–	–	–	1	–	–	–	1	1	1	1	1	1	1	1
DE	–	–	–	–	0.67	0.33	0.33	–	–	0.44	0.17	0.36	0.12	0.36	0.12
DF	–	–	–	–	0.33	0.67	0.33	–	–	0.17	0.44	0.12	0.36	0.12	0.36
EF	–	–	–	–	0.33	0.33	0.67	–	–	0.17	0.17	0.12	0.12	0.12	0.12

To compare this with the contributions approach [9], Table 3 shows the contributions of each study to a comparison in the bridge network. For all comparisons of treatments from different parts of the network the values of contributions differ from those of importances. This is because the contributions approach attributes lower weights to a study when the network distance between the treatments is greater.

**Table 3:** Contributions of each study for the network with a bridge following the contributions approach [9]

	Comparisons														
Study	A:B	A:C	B:C	C:D	D:E	D:F	E:F	A:D	B:D	C:E	C:F	A:E	A:F	B:E	B:F
AB	0.67	0.33	0.33	–	–	–	–	0.11	0.11	–	–	0.07	0.07	0.07	0.07
AC	0.33	0.67	0.33	–	–	–	–	0.33	0.11	–	–	0.22	0.22	0.07	0.07
BC	0.33	0.33	0.67	–	–	–	–	0.11	0.33	–	–	0.07	0.07	0.22	0.22
CD	–	–	–	1	–	–	–	0.44	0.44	0.44	0.44	0.29	0.29	0.29	0.29
DE	–	–	–	–	0.67	0.33	0.33	–	–	0.33	0.11	0.22	0.07	0.22	0.07
DF	–	–	–	–	0.33	0.67	0.33	–	–	0.11	0.33	0.07	0.22	0.07	0.22
EF	–	–	–	–	0.33	0.33	0.67	–	–	0.11	0.11	0.07	0.07	0.07	0.07

Entries in italics differ from those in Table 2

#### A generic triangle

Consider a NMA with three treatments A, B and C and three studies comparing A to B, A to C, and B to C with variances *V*_*AB*_,*V*_*AC*_,*V*_*BC*_. We focus on comparison A:B. Its direct estimate has variance *V*_*AB*_ and its NMA estimate has variance

$$V_{all}(A:B)\! =\! \frac{1}{V_{AB}^{-1} + \left(V_{AC} + V_{BC}\right)^{-1}} \!= \frac{V_{AB} \ (V_{AC} + V_{BC})}{V_{AB} + V_{AC} + V_{BC}}$$

The direct evidence proportion for A:B is

$$\frac{V_{AC} + V_{BC}}{V_{AB} + V_{AC} + V_{BC}}.$$

If one of the studies on the indirect pathway from A to B (say AC) is removed, only direct evidence remains, and we get its importance for comparison A:B as

8 $$ \begin{aligned} p(AC,A:B) &= 1 - \frac{V_{all}(A:B)} {V_{AB}} = 1 - \frac{V_{AC} + V_{BC}}{V_{AB} + V_{AC} + V_{BC}}\\ &= \frac{V_{AB}}{V_{AB} + V_{AC} + V_{BC}}, \end{aligned}  $$

the same if BC is removed. In other words, it does not matter whether we remove AC or BC or which of them has smaller variance, the importance of the two studies is equal and also equal to the indirect evidence proportion. The indirect evidence proportion comes from the *combination of studies* AC and BC and depends on the sum of their variances. This example shows that it does not make sense to add up the importances of all studies. It also challenges the idea of breaking up the indirect evidence proportion into additive parts from each study.

### Real data networks

We now apply our method to the two real data sets presented before.

#### Parkinson’s data

**Fig. 3:**
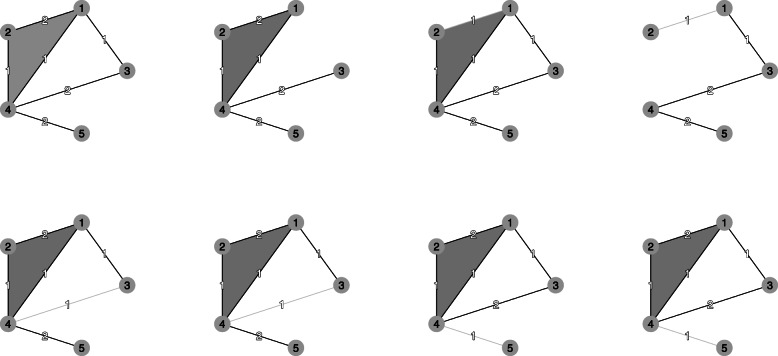
Parkinson’s data (top left panel) with each study removed in turn (other panels). 1 = placebo, 2 = pramipexole, 3 = ropinirole, 4 = bromocriptine, 5 = cabergoline

Figure 3 shows the network graph of the Parkinson’s data [28] (top left panel) and, for each of the seven studies, the effect of removing a single study (study 1: comparison 1:3; study 2: comparison 1:2; study 3: comparisons 1:2, 1:4, 2:4; studies 4,5: comparison 3:4; studies 6,7: comparison 4:5).

The resulting importances from the common effect model are given in Table 4. The last row of the table provides the direct evidence proportions for each comparison.

**Table 4:** Importance of each study for the comparisons in the Parkinson’s data

	Comparisons										
Study	Design	1:2	1:3	1:4	1:5	2:3	2:4	2:5	3:4	3:5	4:5
1	1:3	0.029	**0.531**	0.405	0.364	0.418	0.294	0.264	0.126	0.092	–
2	1:2	**0.756**	0.076	0.121	0.104	0.402	0.379	0.345	0.013	0.009	–
3	1:2:4	**0.244**	0.469	**0.595**	0.553	0.503	**0.617**	0.582	0.126	0.092	–
4	3:4	0.005	0.128	0.101	0.086	0.130	0.064	0.056	**0.535**	0.449	–
5	3:4	0.002	0.061	0.048	0.041	0.062	0.030	0.026	**0.339**	0.266	–
6	4:5	–	–	–	0.178	–	–	0.157	–	0.285	**0.577**
7	4:5	–	–	–	0.105	–	–	0.092	–	0.177	**0.423**
Direct evidence (%)		97.1	53.1	47.4	0.0	0.0	55.8	0.0	87.4	0.0	1.0

Direct evidence is printed in bold. The last row shows the proportion of direct evidence for each comparison. 1 = placebo, 2 = pramipexole, 3 = ropinirole, 4 = bromocriptine, 5 = cabergoline

We see that, not surprisingly, the most important study is the three-arm study 3. It is important not only for comparisons 1:2, 1:4 and 2:4, but also for comparison 1:3 and the indirect comparisons 1:5, 2:3 and 2:5. However, for comparison 1:2, study 2 is even more important, and for comparison 1:3 study 1 is more important. Study 4 (comparison 3:4) is the most important study for both comparisons 3:4 and 3:5, whereas the less precise study 5 (likewise comparing 3:4) is less important for all comparisons. For comparison 4:5, only studies 6 and 7 are important. Study 6 is uniformly more important than study 7 (both comparing 4:5).

Study 1, though not very precise, is surprisingly important for comparison 1:4. The only direct evidence for comparison 1:4 comes from the three-arm study 3 with relatively small precision. The only other path from treatment 1 to treatment 4 goes via treatment 3: study 1 provides comparison 1:3, and studies 4 and 5 both provide comparison 3:4. If study 1 is deleted, this path (1→3→4) breaks down, whereas if either study 4 or 5 is deleted, the other study (4 or 5) remains, and the path still exists. Thus study 1 is more important for comparison 1:4 than studies 4 and 5.

For each direct comparison, we may compare this to the contributions (weights) of each study in a pairwise meta-analysis, given in Table 5. For comparisons solely informed by direct evidence (here comparison 4:5) they agree with the corresponding importances.

**Table 5:** Study weights for pairwise meta-analysis of comparisons in the Parkinson’s data (common effects model)

Comparison	Study	Design	Weight
1:2	2	1:2	0.779
	3	1:2:4	0.221
3:4	4	3:4	0.612
	5	3:4	0.388
4:5	6	4:5	0.577
	7	4:5	0.423

1 = placebo, 2 = pramipexole, 3 = ropinirole, 4 = bromocriptine, 5 = cabergoline

#### Thrombolytic data

Table 6 shows results of our method (random effects model) when applied to the thrombolytic data [8, Tables 3 and 4]. The importance values are not directly comparable to percentage contributions, as they do not add up to 100%. Therefore we find major differences to the percentage contributions given by Riley and others [8]. In all these cases, the importance (as we measure it) exceeds the percentage contribution (as defined in [8]). Particularly, study 17 is the only one including treatment E (it compares B to E), and if it is omitted, E is no more part of the network. Therefore study 17 has maximal importance for comparison A:E, compared to 67.13% contribution following Riley’s method. The three-arm study 1 (A:B:D) is more important for comparisons A:E and A:F than when measured by Riley et al.’s % contribution approach. Also study 18, comparing B:F, has higher importance for comparisons A:B, A:E and A:F than when measured by Riley et al.’s % contribution approach. Again, the importance values may be compared to the study weights in pairwise meta-analyses, given in Table 7 for the random effects model.

**Table 6:** Importance of each study for the comparisons to treatment A for the thrombolytic data, multiplied by 100 for comparison to [8, Table 4]

		Comparisons						
Study	Design	A:B	A:C	A:D	A:E	A:F	A:G	A:H
1	ABD	**82.10**	0.03	**99.50**	59.69	50.56	2.68	0.06
2	ACH	0.30	**59.39**	0.03	0.10	0.07	0.50	**96.81**
3	AC	0.00	**0.31**	0.00	0.00	0.00	0.00	0.05
4	AC	0.00	**0.13**	0.00	0.00	0.00	0.00	0.02
5	AC	0.00	**0.13**	0.00	0.00	0.00	0.00	0.02
6	AC	0.00	**38.20**	0.00	0.00	0.00	0.21	9.20
7	AC	0.00	**0.22**	0.00	0.00	0.00	0.00	0.04
8	AC	0.00	**0.38**	0.00	0.00	0.00	0.00	0.06
9	AC	0.00	**0.29**	0.00	0.00	0.00	0.00	0.05
10	AD	0.04	0.00	**0.50**	0.01	0.01	0.00	0.00
11	AF	15.78	0.00	1.57	5.70	**45.67**	0.11	0.00
12	AG	0.14	0.08	0.01	0.05	0.03	**19.35**	0.01
13	AH	0.00	0.03	0.00	0.00	0.00	0.00	**0.15**
14	AH	0.00	0.04	0.00	0.00	0.00	0.00	**0.26**
15	AH	0.00	0.03	0.00	0.00	0.00	0.00	**0.16**
16	AH	0.00	0.12	0.00	0.00	0.00	0.00	**0.75**
17	BE	0.00	0.00	0.00	100.00	0.00	0.00	0.00
18	BF	14.98	0.00	1.47	5.38	52.79	0.11	0.00
19	BF	0.11	0.00	0.01	0.03	0.67	0.00	0.00
20	BG	0.11	0.02	0.01	0.04	0.02	6.75	0.00
21	BG	0.70	0.15	0.06	0.23	0.16	31.50	0.02
22	BH	0.71	0.08	0.06	0.23	0.16	0.00	0.49
23	BH	0.45	0.05	0.04	0.15	0.10	0.00	0.31
24	CG	0.08	0.09	0.01	0.03	0.02	12.11	0.01
25	CG	0.12	0.13	0.01	0.04	0.03	17.31	0.02
26	CG	0.09	0.09	0.01	0.03	0.02	12.59	0.01
27	CH	0.00	0.13	0.00	0.00	0.00	0.00	0.32
28	CH	0.00	0.07	0.00	0.00	0.00	0.00	0.16
Direct evidence		0.82	0.99	0.98	0.00	0.46	0.19	0.89

Direct evidence is printed in bold. The last row shows the proportion of direct evidence for each comparison in a column. A = streptokinase, B = accelerated alteplase, C = alteplase, D = streptokinase + alteplase, E = tenecteplase, F = reteplase, G = urokinase, H = anistreptilase

**Table 7:** Study weights for the thrombolytic data (random effects model)

Comparison	Study	Design	Weight (%)
A:C	2	ACH	59.93
	3	AC	0.32
	4	AC	0.13
	5	AC	0.13
	6	AC	38.59
	7	AC	0.22
	8	AC	0.39
	9	AC	0.30
A:D	1	ABD	99.49
	10	AD	0.51
A:H	2	ACH	98.52
	13	AH	0.17
	14	AH	0.29
	15	AH	0.17
	16	AH	0.85
B:F	18	BF	68.54
	19	BF	31.46
B:G	20	BG	17.79
	21	BG	82.21
B:H	22	BH	61.26
	23	BH	38.74
C:G	24	CG	28.83
	25	CG	41.19
	26	CG	29.99
C:H	2	ACH	98.77
	27	CH	0.81
	28	CH	0.42

A = streptokinase, B = accelerated alteplase, C = alteplase, D = streptokinase + alteplase, E = tenecteplase, F = reteplase, G = urokinase, H = anistreptilase

**Fig. 4:**
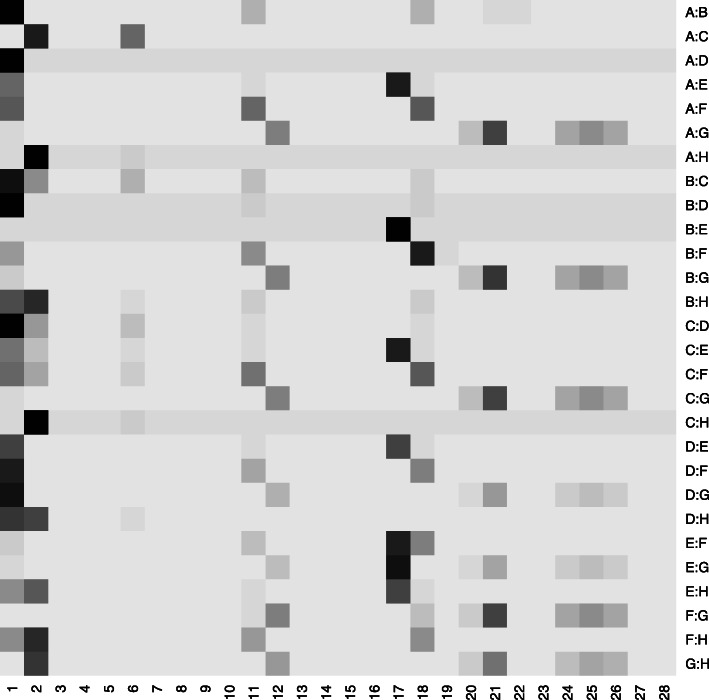
A grayscale heatmap of importances for thrombolytic data. Darker colors represent greater importance of a study (column) for a comparison (row)

A visualization as a heatmap is shown in Fig. 4. Dark colors mean that a study (in the column) is important for the comparison in the row. It is not surprising that studies 1 and 2, both three-arm studies with over 40.000 patients each, are at the top level of importance for most, but not all comparisons. As mentioned before, study 17 (B:E) is important for all comparisons with E, study 18 (B:F) for all comparisons with F, and study 21 (B:G) for all comparisons with G.

## Discussion

In two-stage pairwise MA, the pooled effect estimate is a weighted mean of the study-specific estimates. Relative study weights (for example inverse variance weights) can be defined and interpreted as proportions or percentages, adding to 100%. Notably, inverse variance weights are treated as if they were fixed, though they are estimates of random variables, which has been criticized [31]. Most existing approaches to generalize the concept of ‘weights’ to NMA aim to define study contributions that can be represented as proportions or percentages, like in pairwise MA. At least, it is possible to quantify the proportion of direct and indirect evidence for each NMA estimate, as outlined in the “Methods” section.

Papakonstantinou and others showed that entries in a hat matrix row can be interpreted as a flow through the network where different signs indicate the direction of the flow [9]. Consequently, it makes little sense to add the values of the entries of the hat matrix, as the direction is embedded. In the analogy of a flood, it is always the same water we see in all these coefficients.

### Other possible generalizations of the direct evidence proportion

The idea of comparing variances also underlies the ‘Borrowing of strength’ (BoS) measure, developed in the more general framework of multivariate meta-analysis [7, 8]. With respect to interpretation, Copas and others distinguish between ‘Direct interpretation’ (which corresponds to our interpretation 1), ‘Add-one-in interpretation’ and ‘Leave-one-out interpretation’ (which both refer to our interpretation 3) [20].

Different interpretations suggest different ideas of generalizing the *p*(*c*). A generalization of interpretation 1 (i.e., splitting direct and indirect contributions) aims to determine the contribution of each comparison (or, alternatively, of each study) to a given NMA estimate, such that these contributions add up to 100% (‘percentage contributions’). This means splitting 1−*p*(*c*), the proportion of indirect evidence, further into parts coming from different comparisons or studies, as done by [9]. Starting from interpretation 2 would mean looking for a quantity that describes the proportion to which the variance of a given direct estimate at hand decreases by *adding indirect evidence* from a particular other study. However, adding another comparison to a given direct comparison makes only sense if the enlarged network is connected, that is if the new study and the comparison in question have treatments in common or if further studies are added. Thus, interpretation 2 does not seem to be a good starting point for generalization. Therefore, we focus on a generalization that is motivated by interpretation 3.

### The concept of importance for the variance

Our concept of study importance does not start from the hat matrix, but interprets the importance of a study to a comparison as the relative reduction of the variance of the estimate when adding the study to the network. We refrain from requesting the values to sum up to 1. For example, a study can be essential for a comparison (like study 17 in the thrombolytic data for all comparisons involving treatment E), thus providing an importance of 1, but other studies may be also (or even equally) important. For instance, in the chain or the circle example, all studies on a path are equally important for the comparison of the path’s ends.

### Versatility of the importance approach

We emphasize that our definition does not rely on, and actually is not restricted to, inverse variances. This is because no variance estimate of a direct comparison enters definition (1). Rather, we define importances as ratios of estimated variances from two different NMAs which could be based on any method, including one-stage approaches or specific methods for binary outcomes. In Additional File 1, we show how to use the Mantel-Haenszel (MH) method to estimate importances both in a pairwise meta-analysis and a network meta-analysis using the recently developed MH method for NMA [26]. This works also in a Bayesian framework.

### Importance and contribution

For pairwise MA there is no ambiguity: the contributions (weights) of all studies add to 1, however they were determined. Likewise, in NMA the direct evidence can be broken down into percentage weights. The division into direct and indirect contributions in NMA (which add to 1) is also possible. However, it is the breakdown of percentage weights for the *indirect* evidence that does not work. Therefore, for the more general situation, we use ‘importance’ instead of ‘contribution’, because these two words have different connotations. Coming back to the example of the chain, the importance of each of the studies connecting A to D is 1, meaning that each of these studies is needed for comparing A to D. By contrast, the concept of ‘contribution’ by Papakonstantinou and others [9] accounts for the fact that though all these studies are *necessary*, none of them alone is *sufficient* for comparing A and D. Therefore for this example they divide 1 by the number of linking studies, which is three, leading to a contribution of 1/3 for each study in the path. However, this approach is not strictly deterministic, as demonstrated in [9, Supplementary File 3].

### Combinations of studies matter

The importance of a study for a comparison must be seen in combination with other studies. Possible extensions could be to define the importance of combinations of studies, or the importance of a single patient in a study to a NMA estimate. We emphasize that the importance of a study is always conditional on the other studies being included in the network. There is an analogy to a multivariable regression model: If the association of each regressor (covariate) *x*_*i*_ with the dependent variable is considered in isolation, the proportion of explained variance of the dependent variable is given by its coefficient of determination, $r_{i}^{2},$ which is bounded between 0 and 1. When considering more than one covariate, it does not make sense to consider their $r_{i}^{2}$ values separately (or even to add them). The proportion of explained variance for the multivariable model depends on the selected variables and their correlation structure. While the goodness of fit for the model can be measured by its coefficient of determination, it is far less clear what is meant by (or how to measure) the ‘contribution of each variable to the outcome’, let alone in percent. This is also true for the regression coefficients, as each of them depends on the selected model. In our view, the situation in NMA is similar. Indeed, NMA can be written as a meta-regression model, and so has the same issues of defining the contribution of each variable in isolation.

### Random effects model

For the random effects model, the omission of a study may decrease some variances, if $\hat \tau ^{2}$ is not fixed. For example, for a circular network (Fig. 2, left panel) leaving out a study always leads to zero inconsistency, because the resulting network is free of loops. If inconsistency is large for the primary network, the variances of all estimates decrease for the subnetworks, resulting in negative importances. In principle, we could accept negative importances: an obvious interpretation would be that a study with negative importance ‘disturbs’ the network. However, we prefer to fix $\hat \tau ^{2}$ to the heterogeneity variance estimate from the full network, in line with the definition of the random effects weights for pairwise MA. This means that for the random effects model information of the full network enters the estimation for all subnetworks. Moreover, as $\hat \tau ^{2}$ does not only depend on the variances, but also on the treatment effect estimates of all studies, information on the treatment effect estimates enters the importance values in the random effects model (as do the random effects weights in a pairwise MA). This issue is covered in detail in [7] and led Copas and others to focus mainly on common effect models [20].

### Impact on the variance or on the effect estimates?

The importance of a study in a network MA can be considered from different aspects. In this article, we follow [6, 8, 9], focussing on the impact of a study on the *variances*. This type of approach generalizes the inverse variance weights in pairwise common effects MA and, like these, ignores the impact of a study on the actual *treatment effect estimates*. A study may be important because of its high precision, but this does not necessarily impact the size or direction of the effect estimates. For example, there is a marked inconsistency between the direct and the indirect estimates for comparison B:H in the thrombolytic data example: the direct effect for B:H deviates from the indirect effect, which is mainly driven by the large studies 1 and 2. Such deviations are not the focus of variance-based methods. For those mainly interested in treatment effects, we point to approaches to identify influential studies (including ‘outliers’) which impact the effect estimates [2–5]. These concepts do not rely exclusively on the structure of the network and the variances of the studies, but also account for their effect estimates and the extent to which they are consistent with estimates from other studies. These methods differ from the variance-based methods in their aims.

## Conclusion

We propose to measure the importance of a study for a comparison in a NMA as the relative reduction of the variance of the estimate when adding the study to the network, or, equivalently, the relative loss of the precision when the study is left out. This works with both two-stage and one-stage NMA, also in a Bayesian framework. For pairwise MA, importances reduce to the usual inverse variance weights. Importances are values between 0 and 1 and cannot be expected to add up to 1. An importance of 1 means that the study is an essential link of the pathway in the network connecting one of the treatments with the other. This may possibly also hold for multiple studies on a pathway. The importance of a study for a comparison depends on the network structure and on other studies on the paths from one node in the network to another. Accordingly, our variance-based measure provides some insight into the network structure. For the common effect model, importances, like the weights in pairwise MA, are not informative with respect to the size, direction, or risk of bias of the effect estimates. This is different for the random effects model where the effect estimates influence the importances via $\hat \tau ^{2}$ like the random effect weights in pairwise MA.

## Supplementary information

**Additional file 1** R code to produce all analyses described in this paper.

**Additional file 2** Plots resulting from running all commands in Additional File 1.

**Additional file 3** Thrombolytic data in csv format (can be viewed as an Excel file).

## Data Availability

All data in this publication are either provided in the manuscript or in the additional files. All R code needed to reproduce the analyses in this publication is provided in Additional file 1.

## Abbreviations

BoS: borrowing of strength
CINeMA: confidence in network meta-analysis
MA: meta-analysis
NMA: network meta-analysis
SIDE: separating indirect and direct evidence
SIDDE: separating indirect from direct design evidence
