# Supplementary material for: The statistical importance of a study for a network meta-analysis estimate
Source: BMC Med Res Methodol. 2020 Jul 16;20:190. doi: 10.1186/s12874-020-01075-y (PMC7386174; doi:10.1186/s12874-020-01075-y)
Supplement: Supplementary file 2 — Additional file 2 Plots resulting from running all commands in Additional File 1. [file 12874_2020_1075_MOESM2_ESM.pdf]

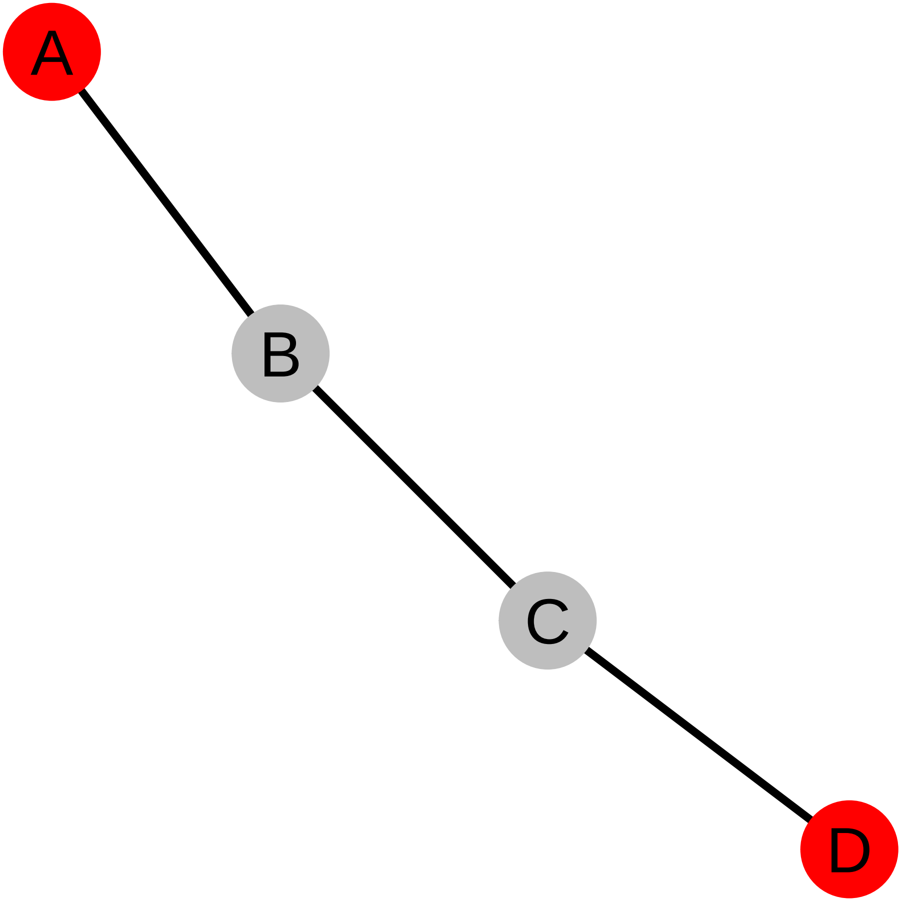
A
B
C
D

Study removed: AB

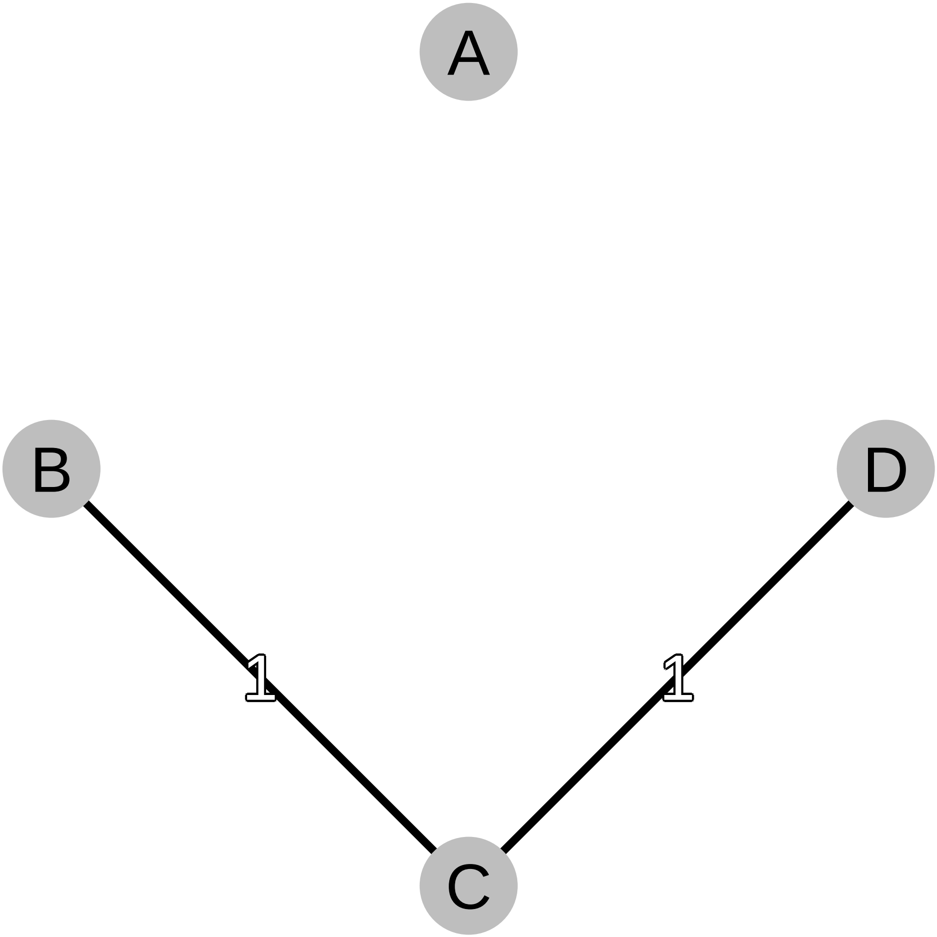

Comparison: 'A:B'

Study removed: BC

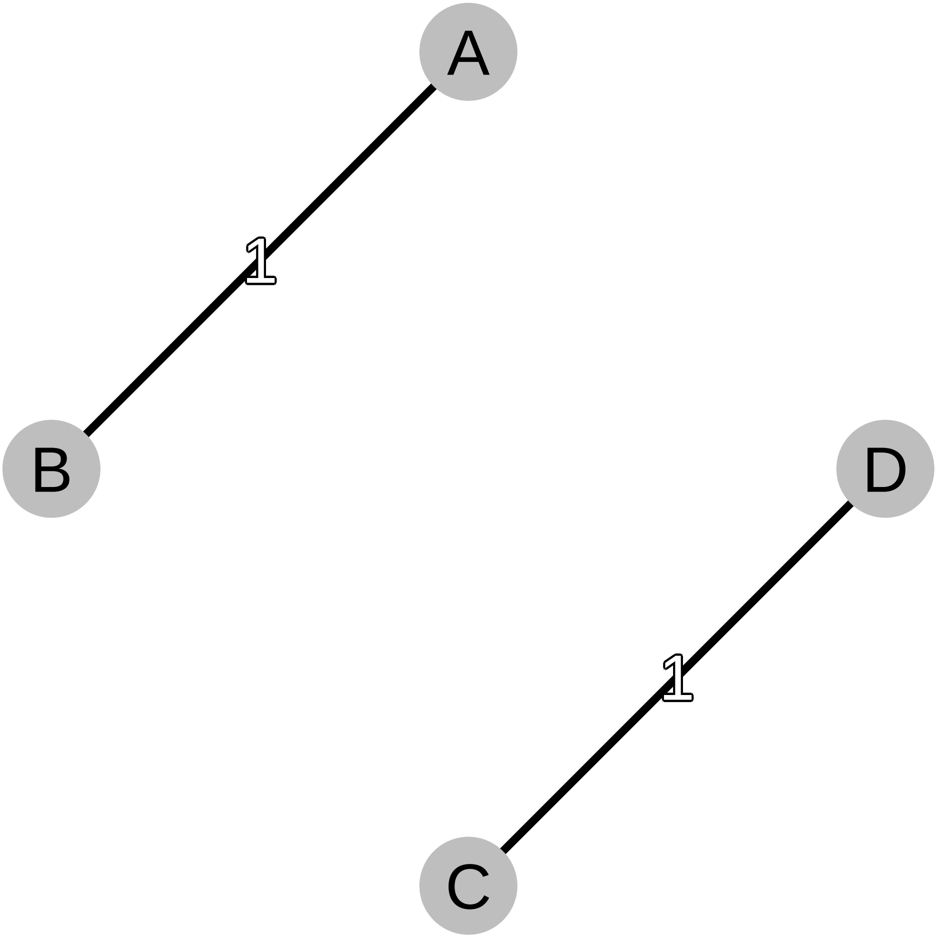

Comparison: 'B:C'

Study removed: CD

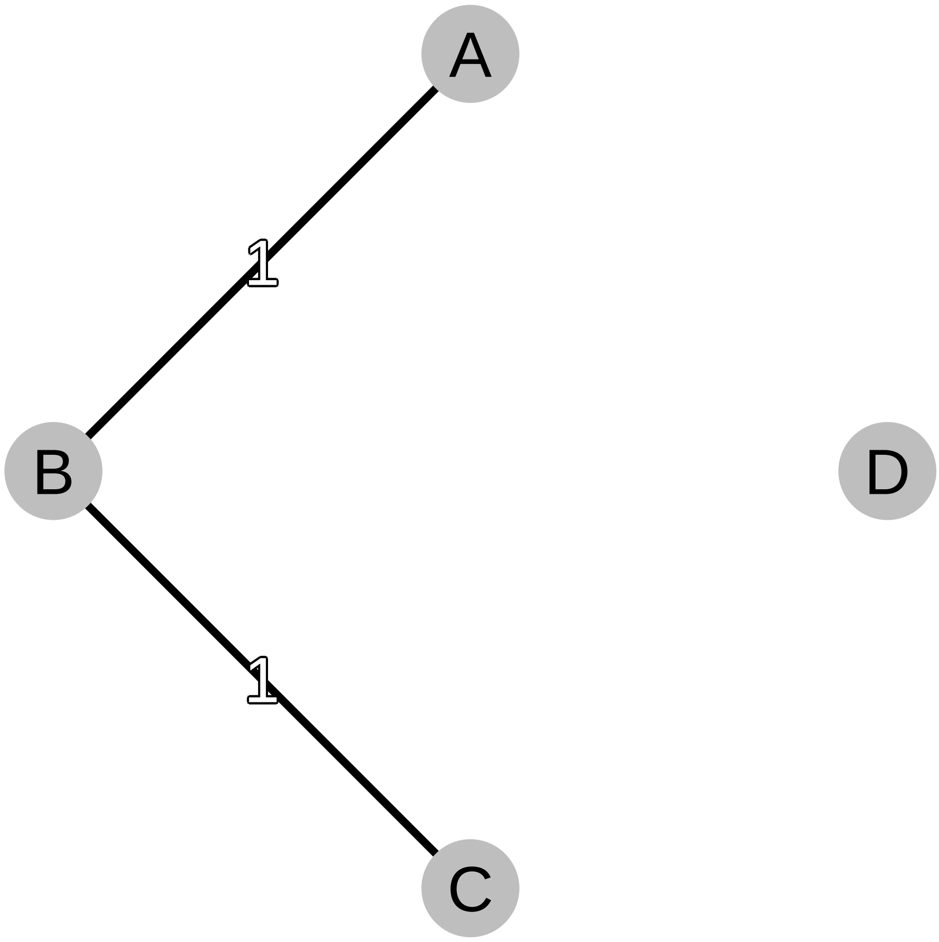

Comparison: 'C:D'

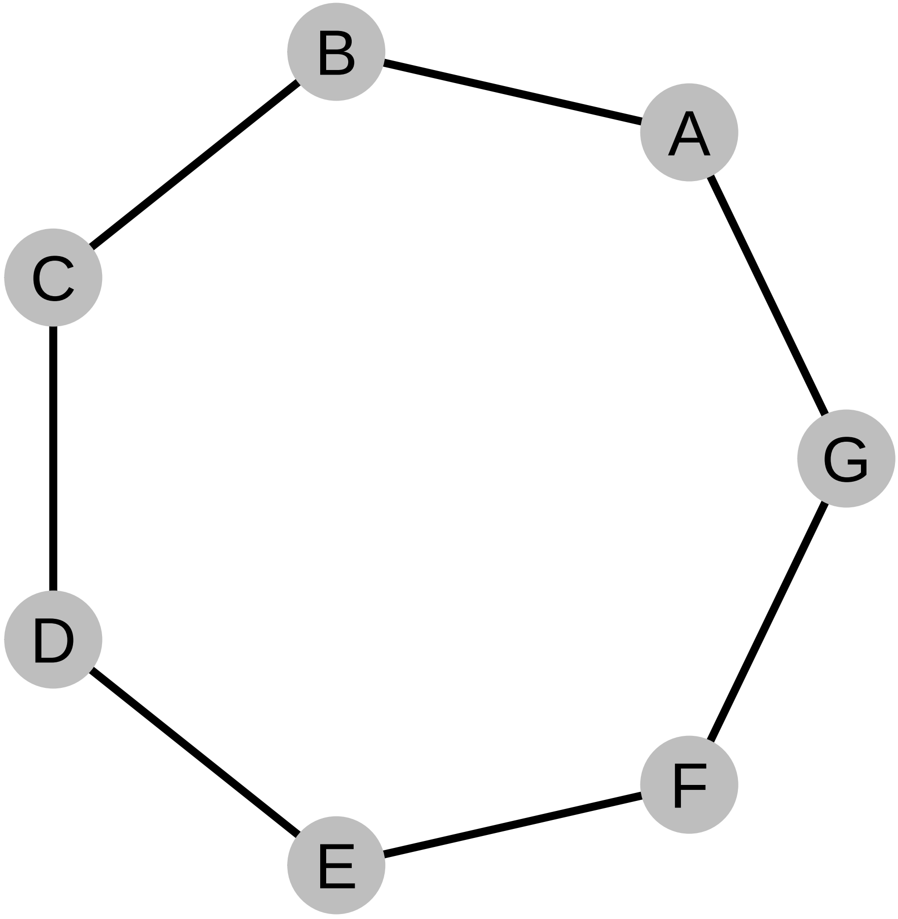
B
A
C
G
D
F
E

Study removed: AB

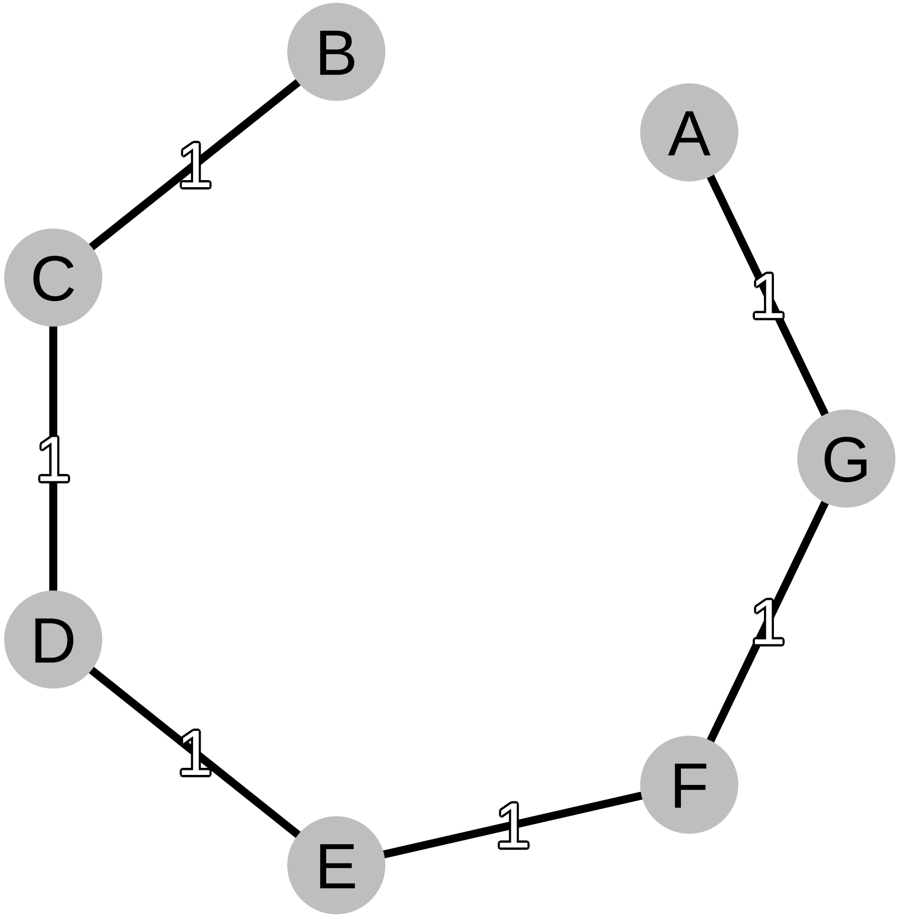

Comparison: 'A:B'

Study removed: BC

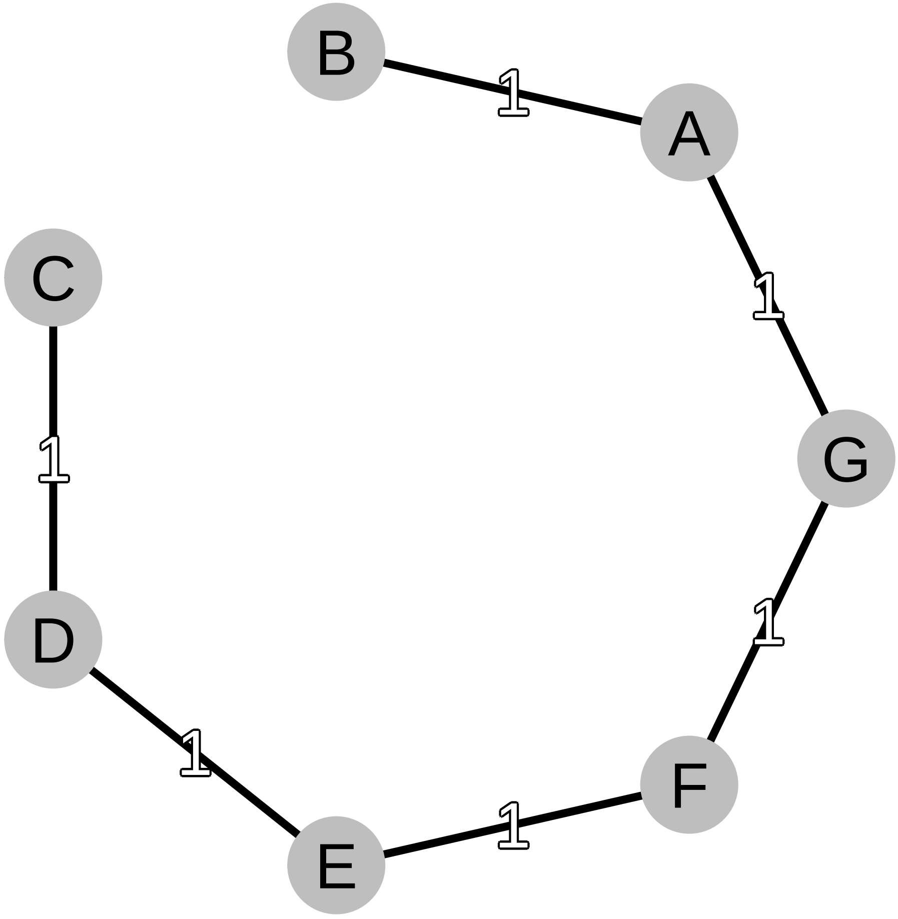

Comparison: 'B:C'

Study removed: CD

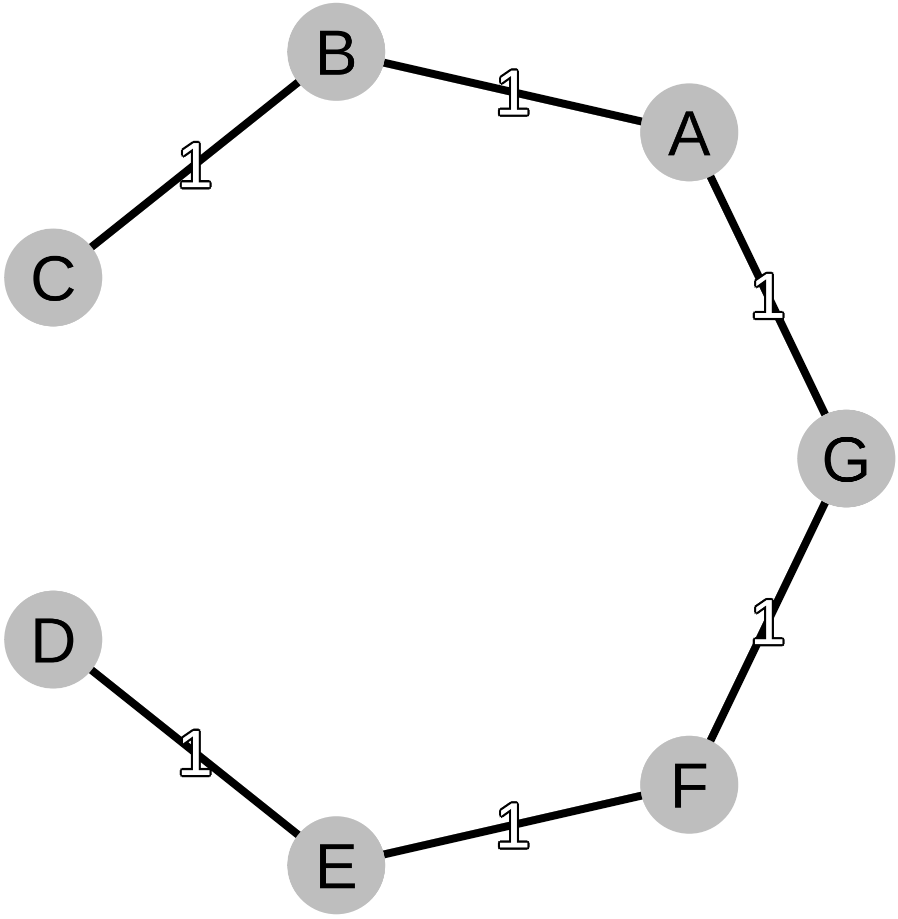

Comparison: 'C:D'

Study removed: DE

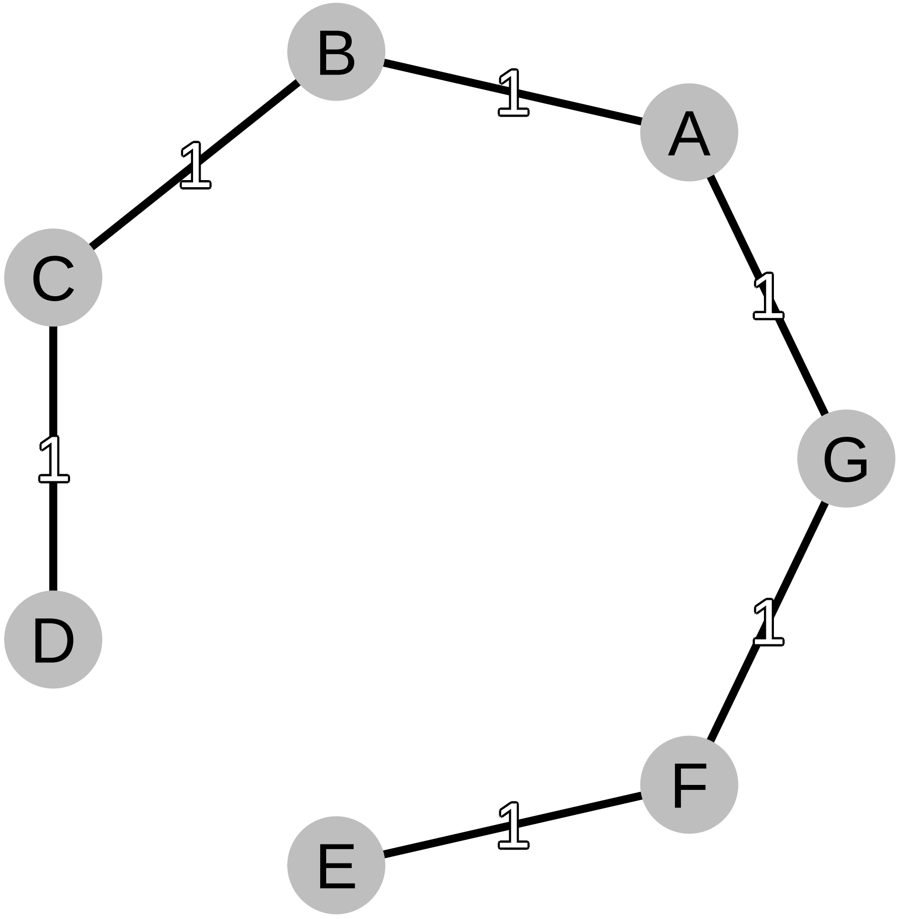

Comparison: 'D:E'

Study removed: EF

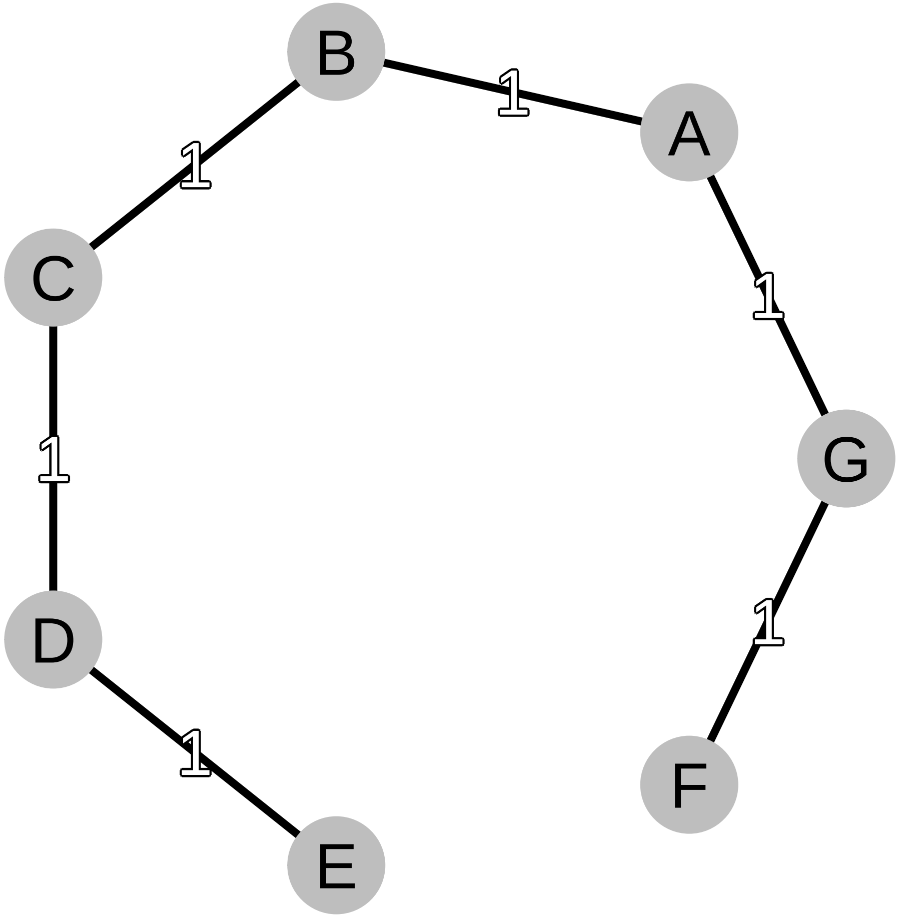

Comparison: 'E:F'

Study removed: FG

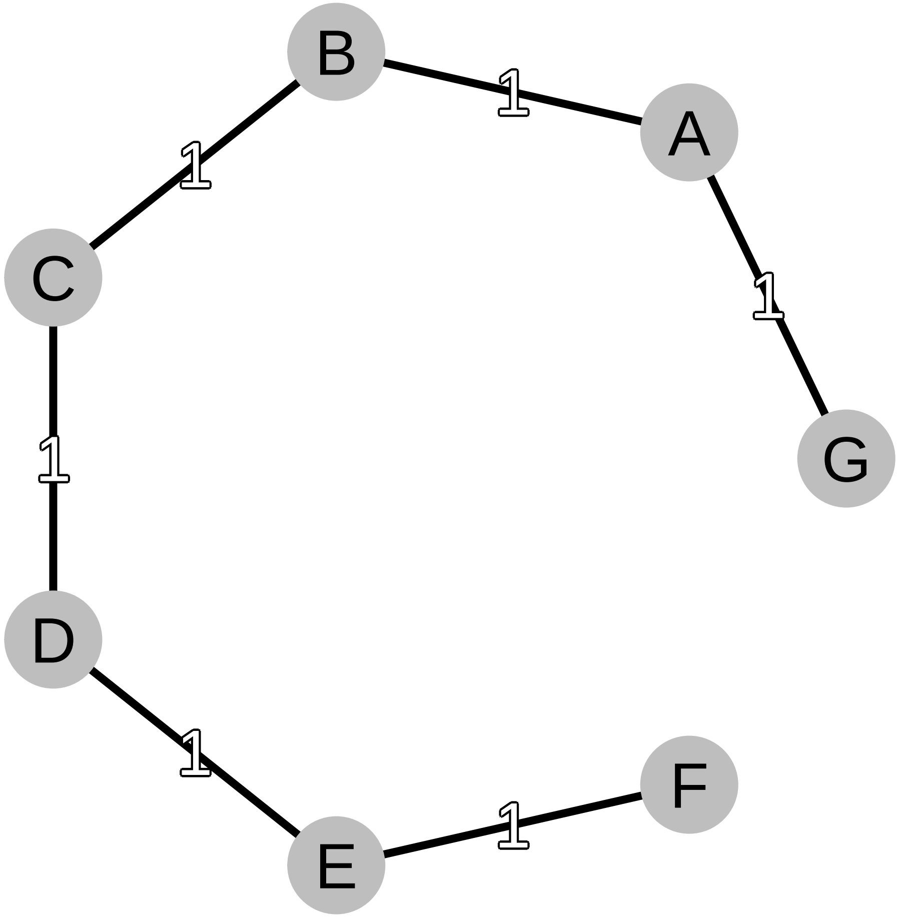

Comparison: 'F:G'

Study removed: GA

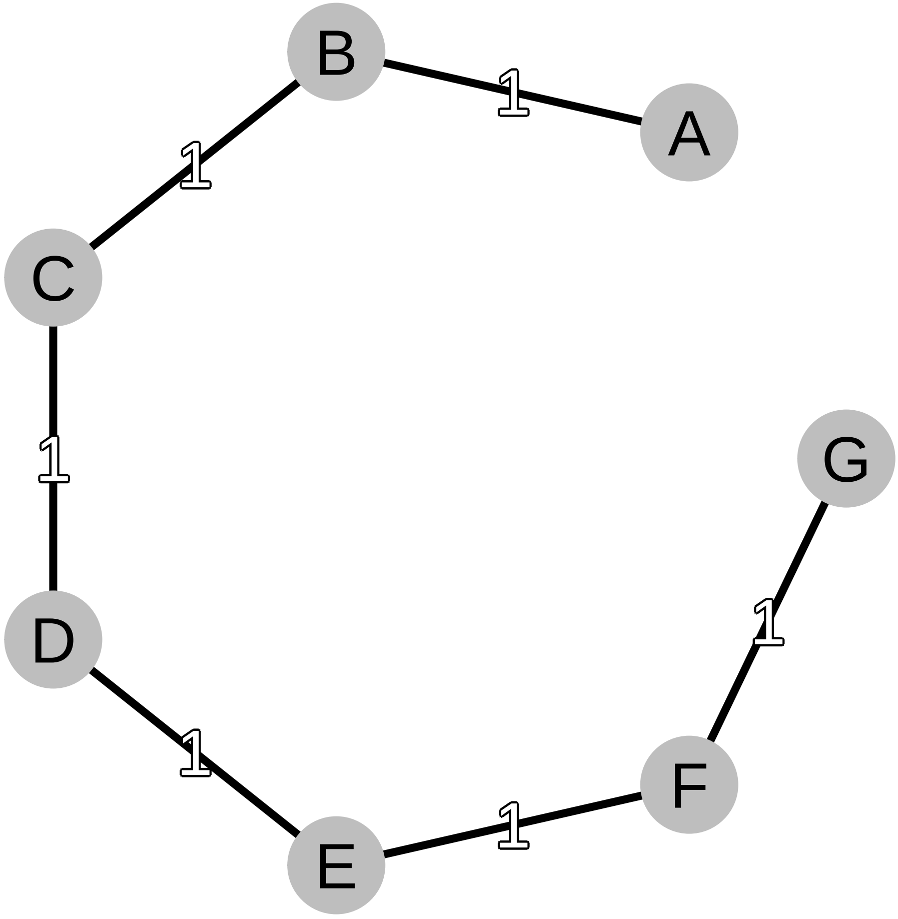

Comparison: 'A:G'

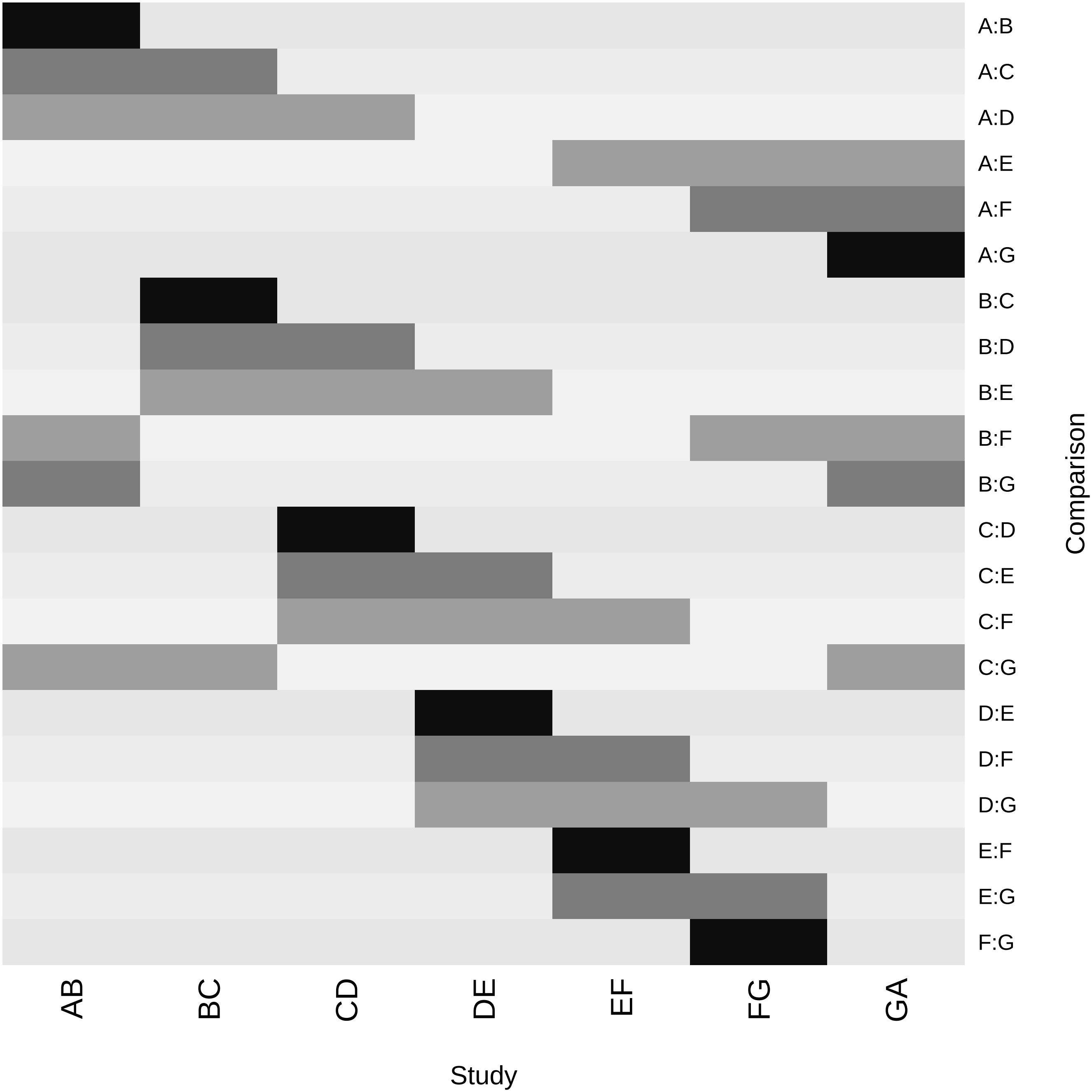

A:B
A:C
A:D
A:E
A:F
A:G
B:C
B:D
B:E
B:F
B:G
C:D
C:E
C:F
C:G
D:E
D:F
D:G
E:F
E:G
F:G
Comparison
AB
BC
CD
DE
EF
FG
GA
Study

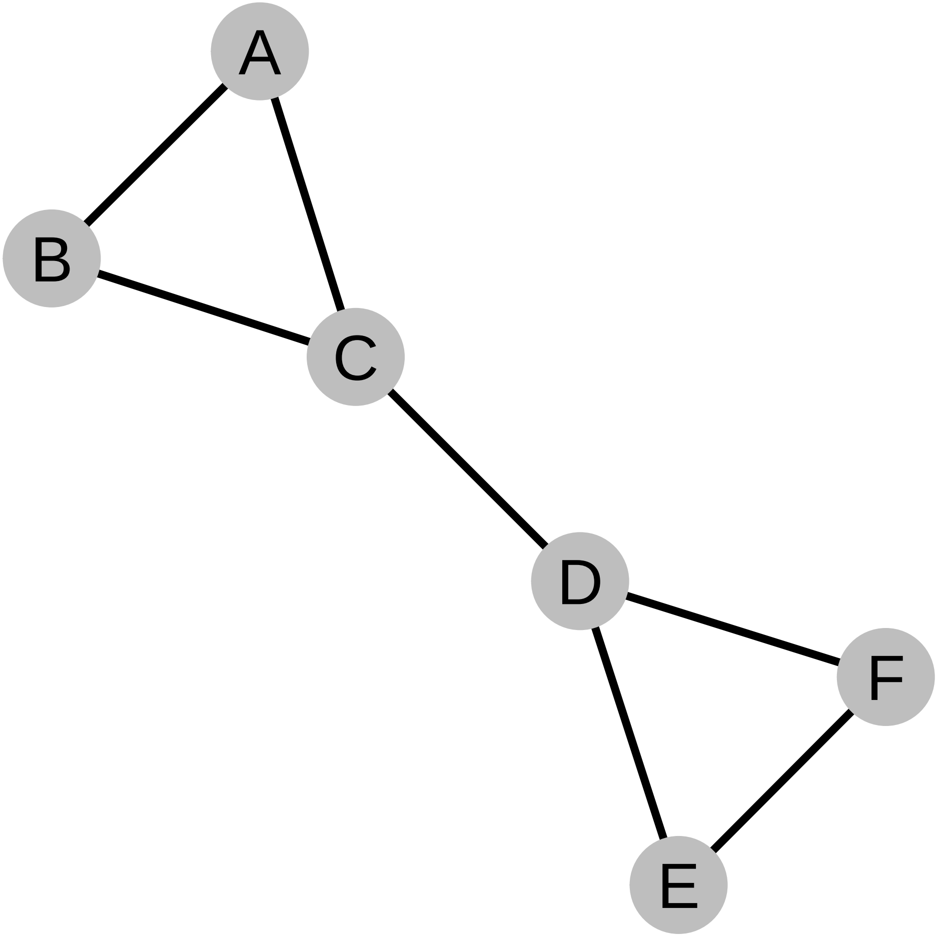
A
B
C
D
E
F

Study removed: 1

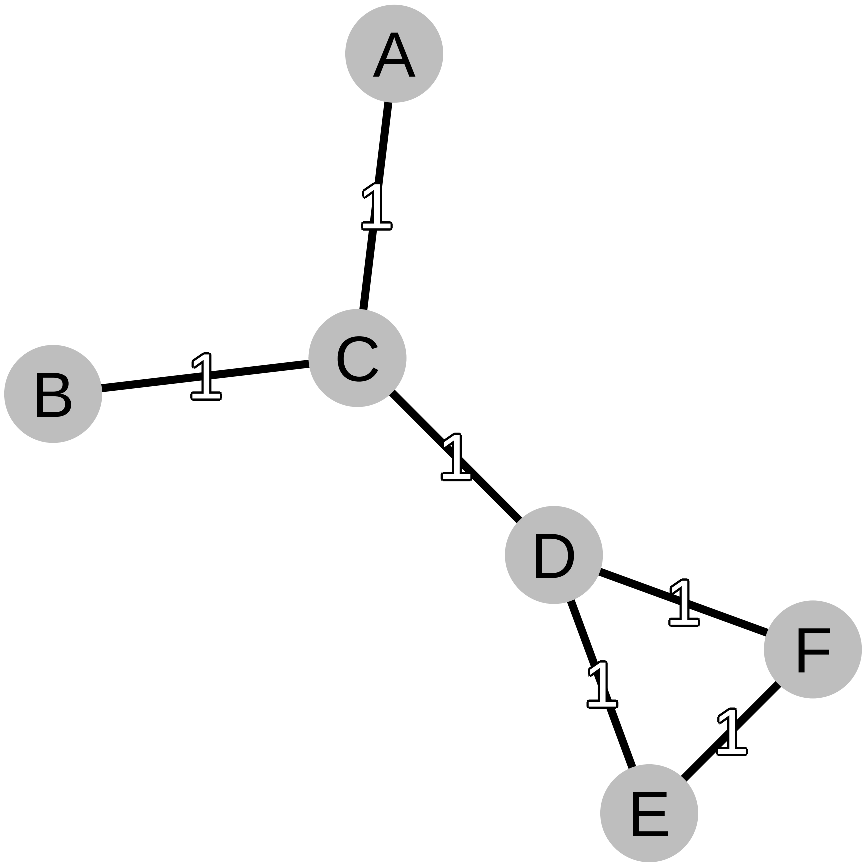

Comparison: 'A:B'

Study removed: 2

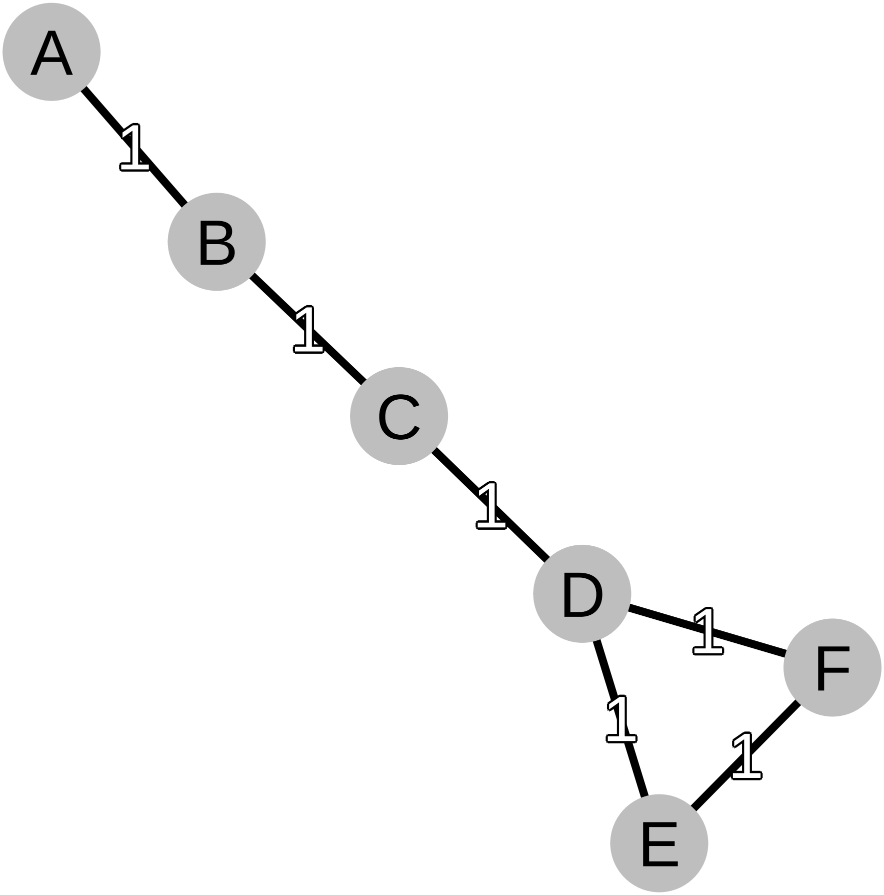

Comparison: 'A:C'

Study removed: 3

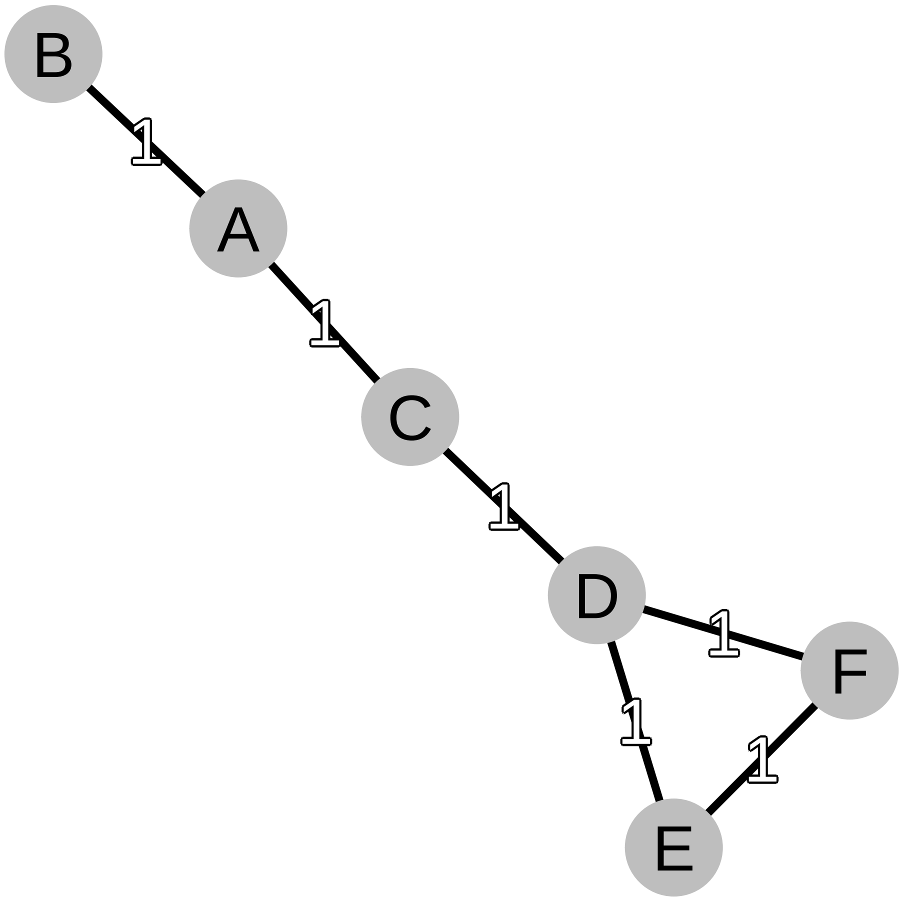

Comparison: 'B:C'

Study removed: 4

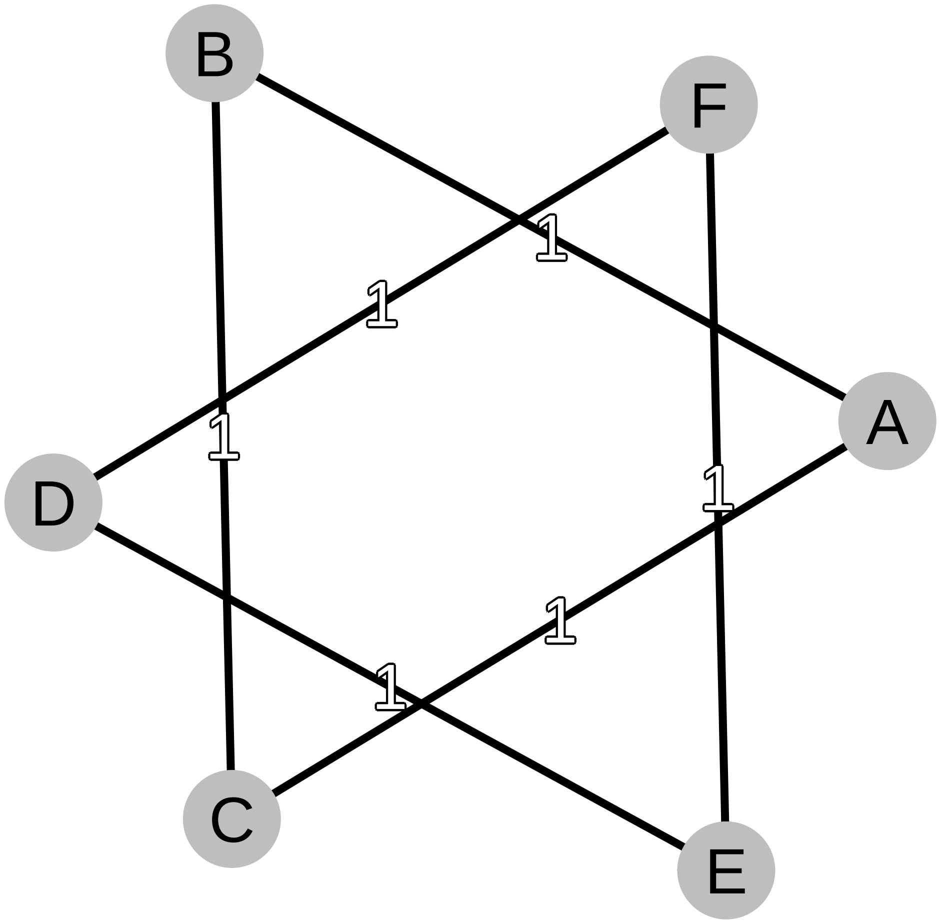

Comparison: 'C:D'

Study removed: 5

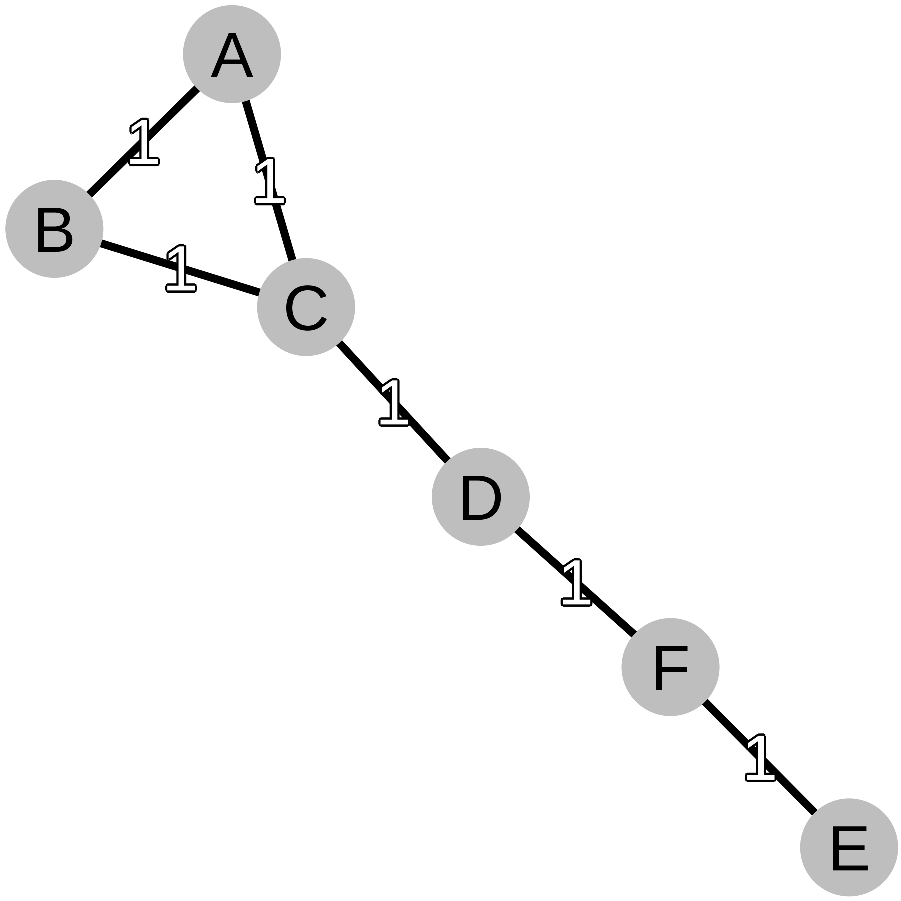

Comparison: 'D:E'

Study removed: 6

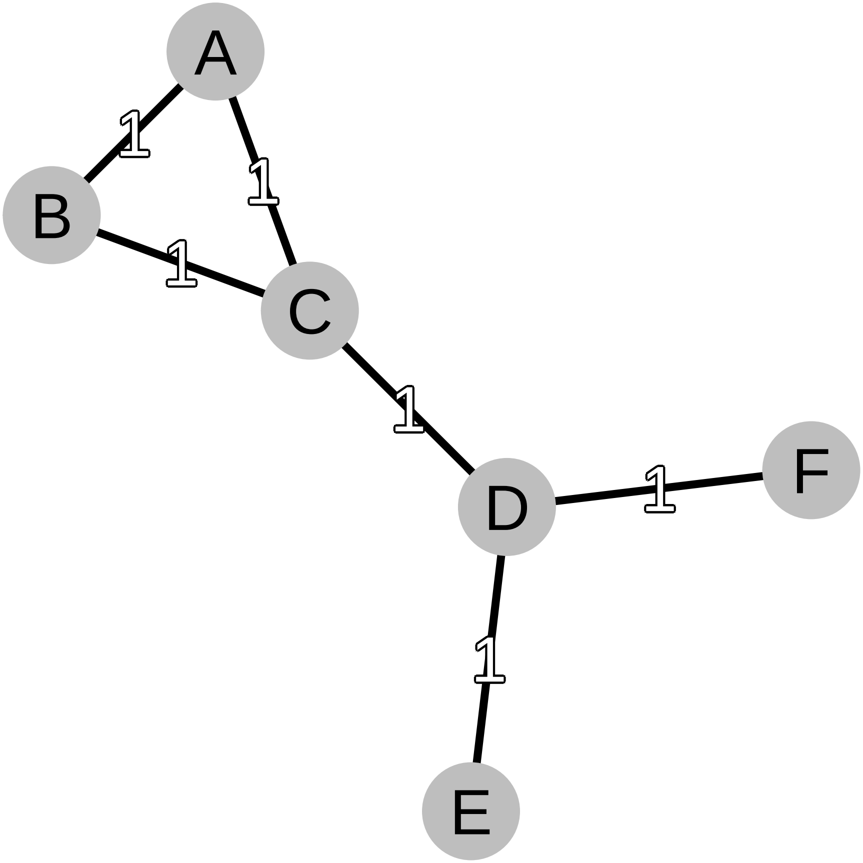

Comparison: 'E:F'

Study removed: 7

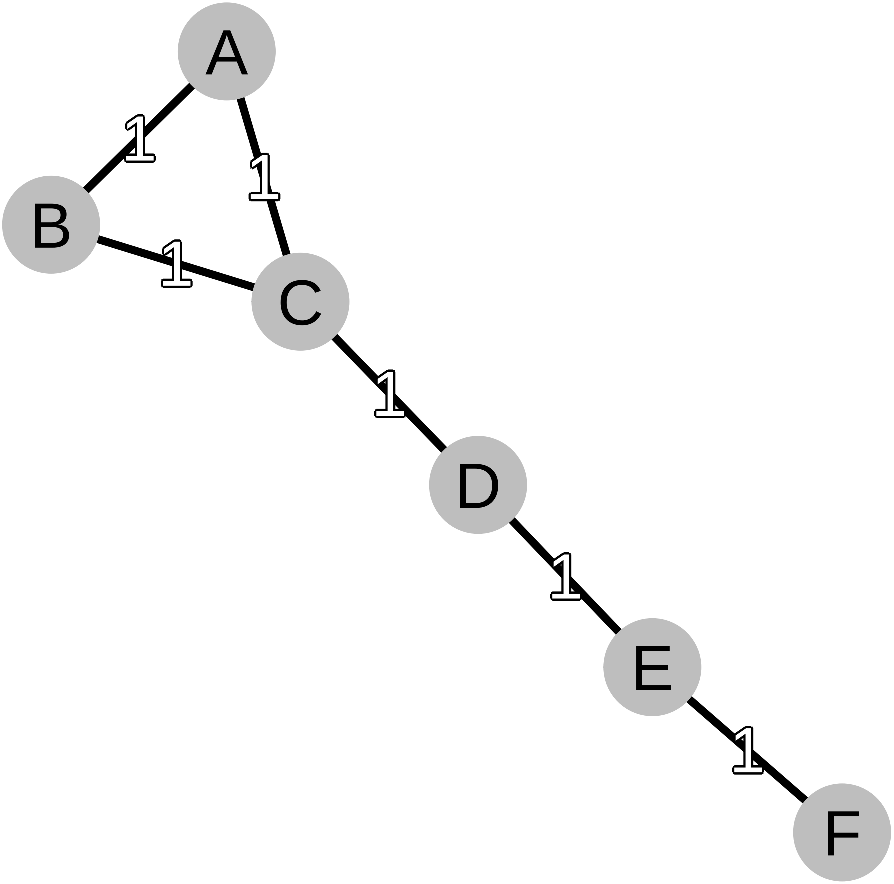

Comparison: 'D:F'

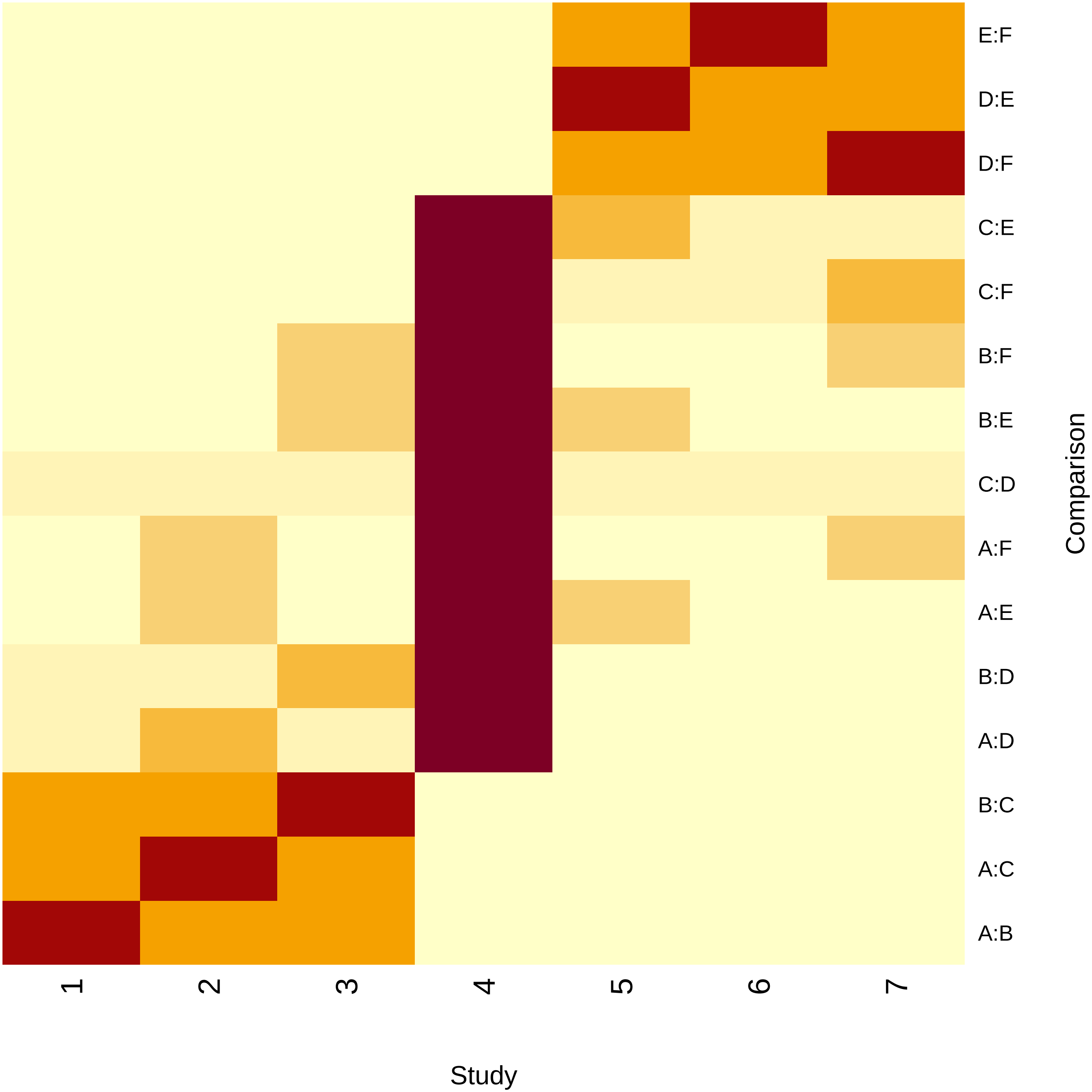
E:F
D:E
D:F
C:E
C:F
B:F
B:E
C:D
A:F
A:E
B:D
A:D
B:C
A:C
A:B
Comparison
1
2
3
4
5
6
7
Study

Full network

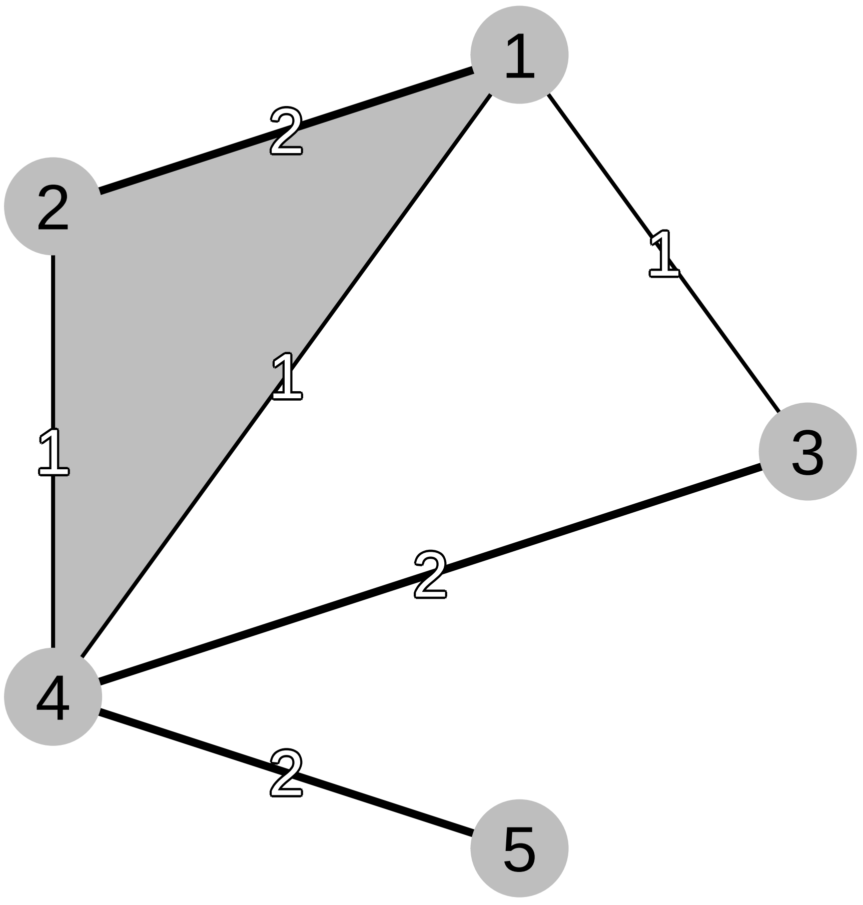

Study removed: 1

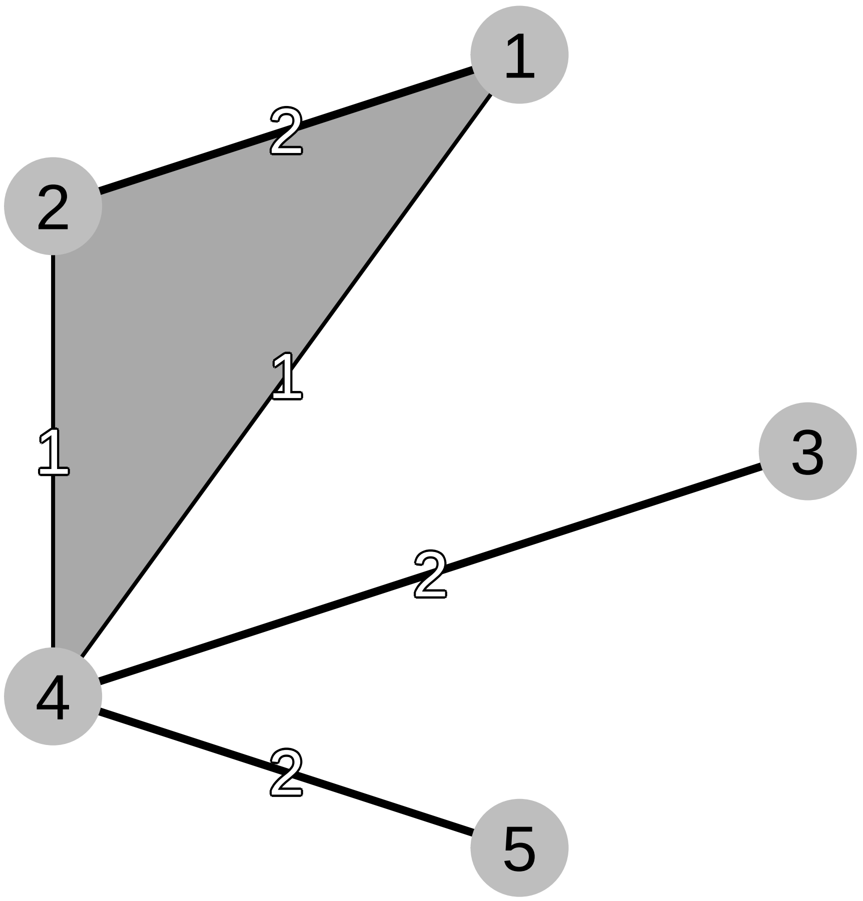

Comparison: '1:3'

Study removed: 2

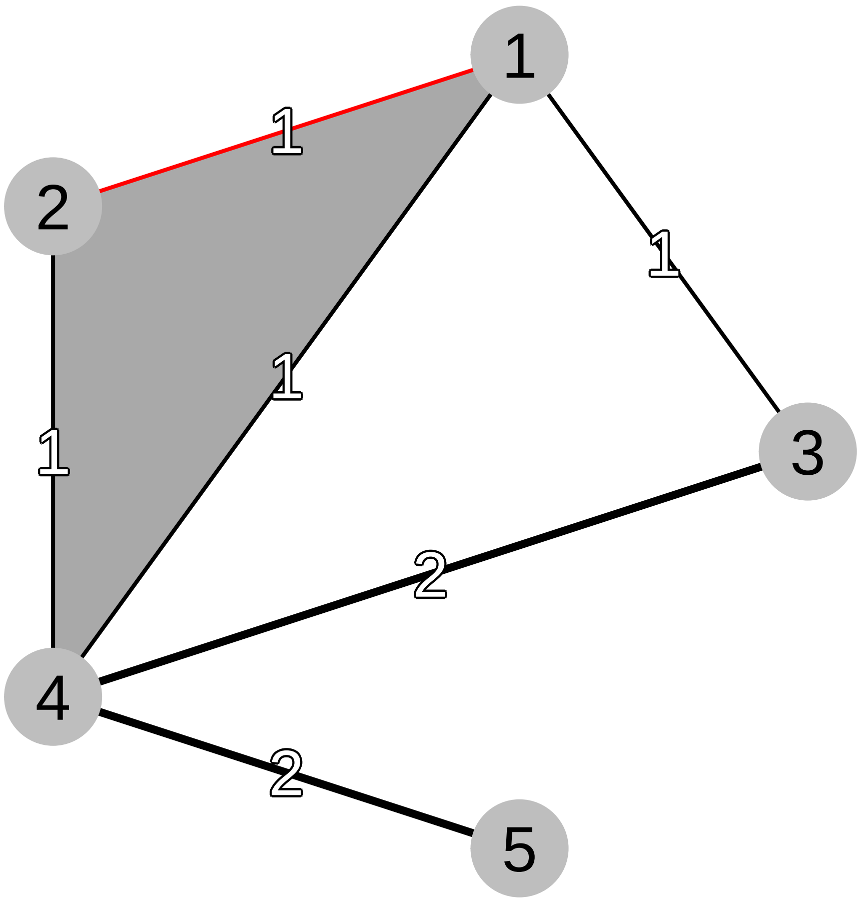

Comparison: '1:2'

Study removed: 3

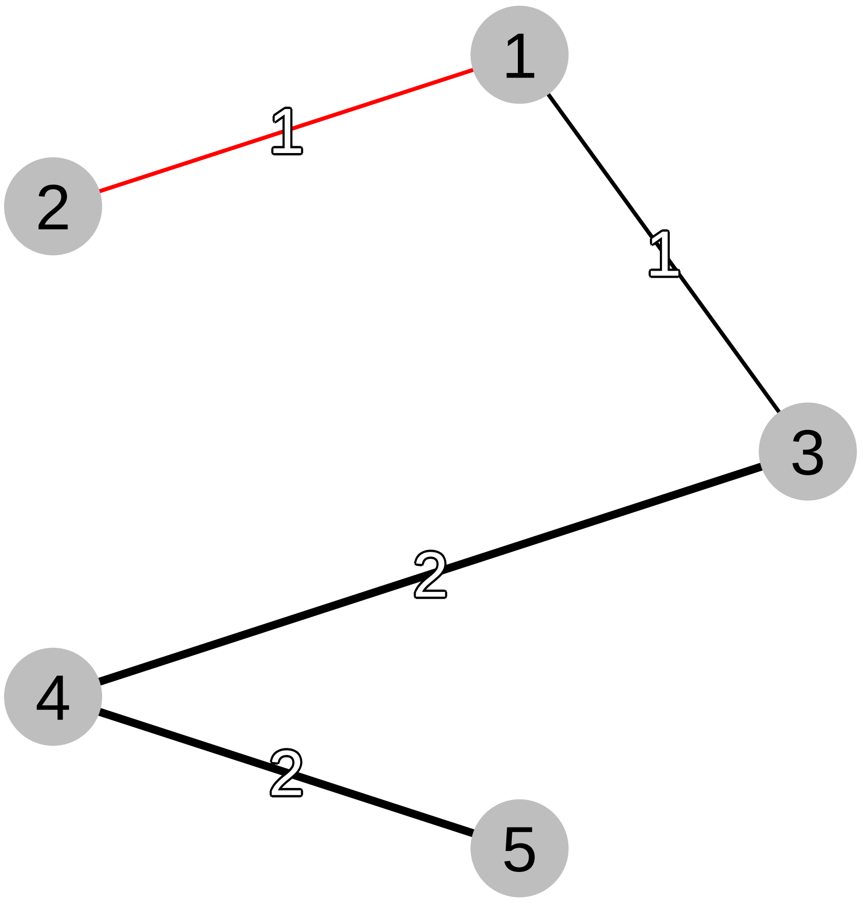

Comparisons: '1:2', '1:4', '2:4'

Study removed: 4

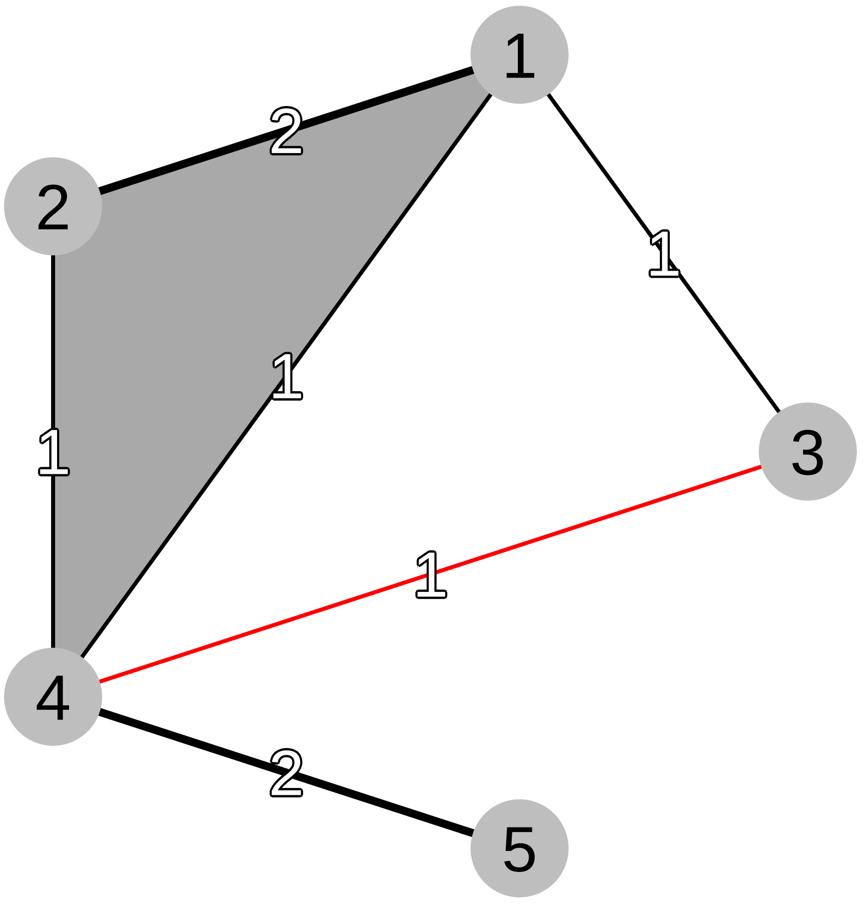

Comparison: '3:4'

Study removed: 5

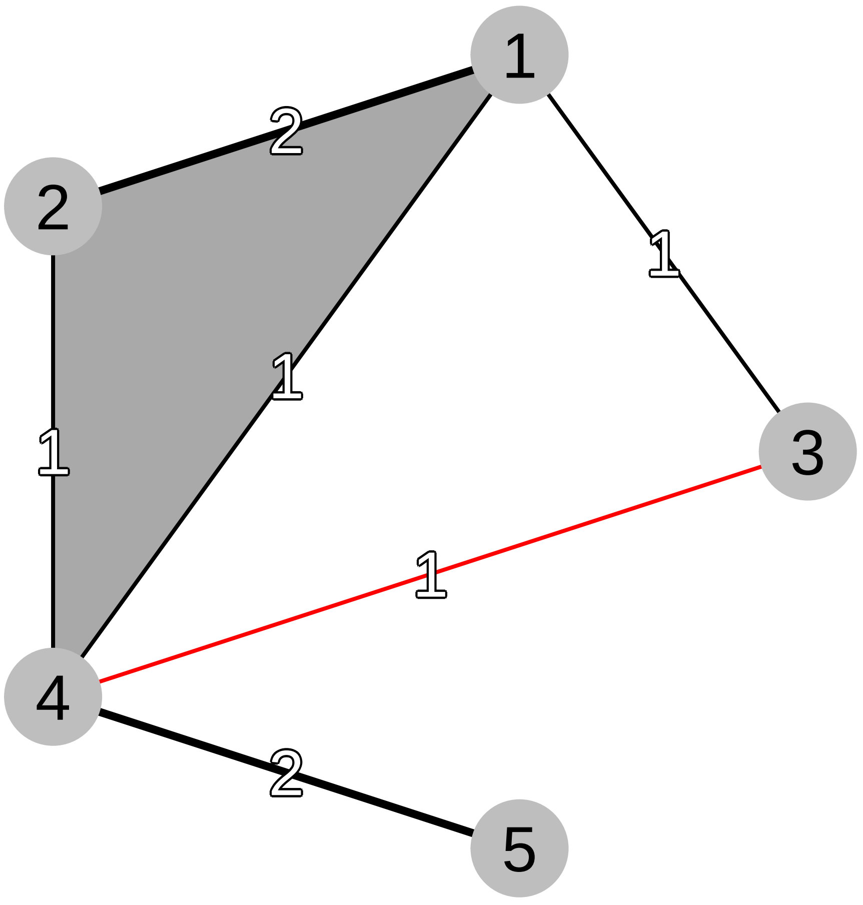

Comparison: '3:4'

Study removed: 6

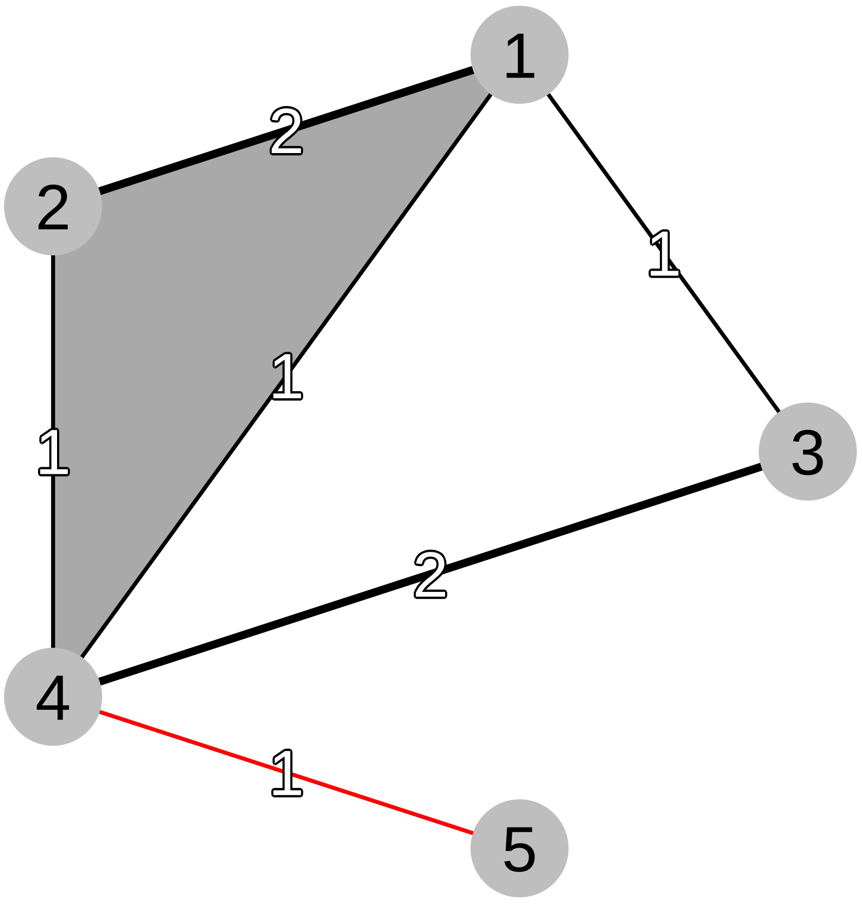

Comparison: '4:5'

Study removed: 7

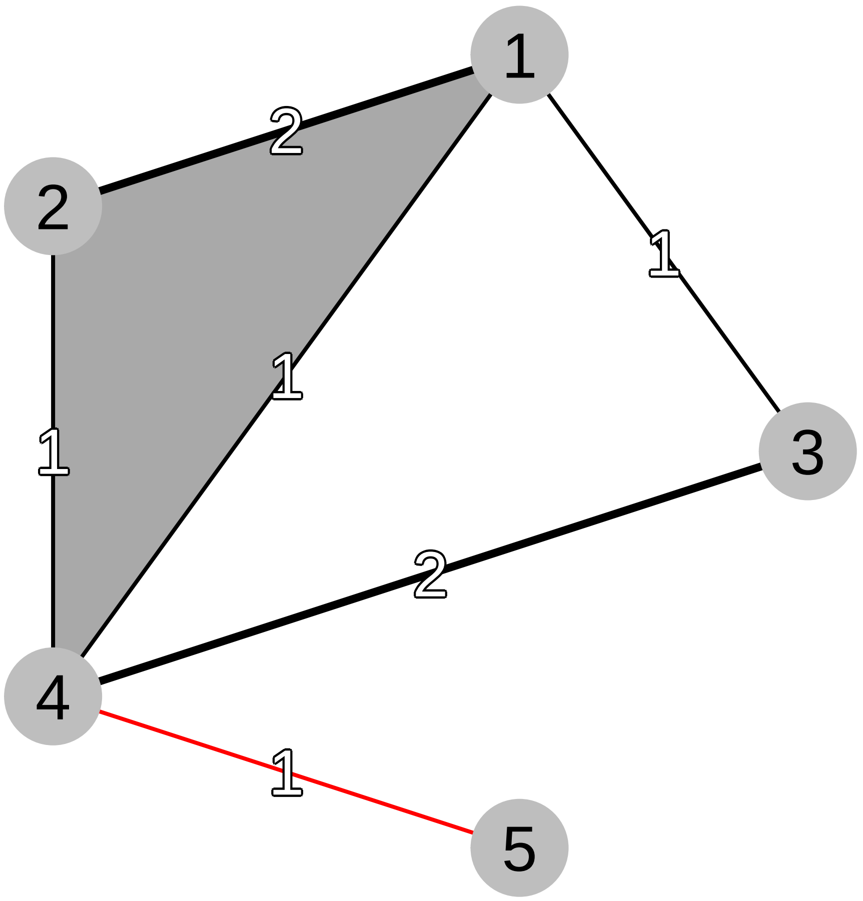

Comparison: '4:5'

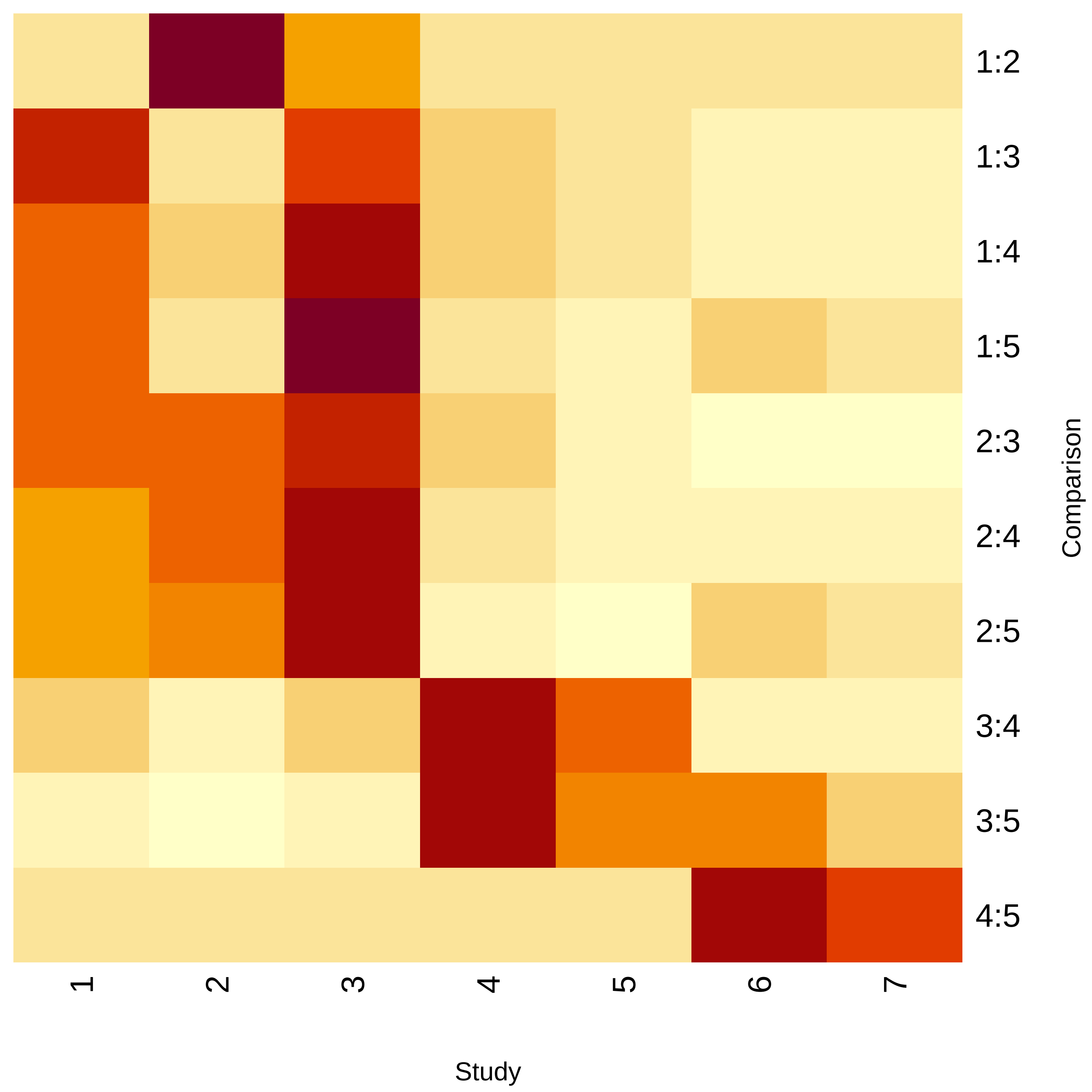
1:2
1:3
1:4
1:5
2:3
2:4
2:5
3:4
3:5
4:5
Comparison
1
2
3
4
5
6
7
Study

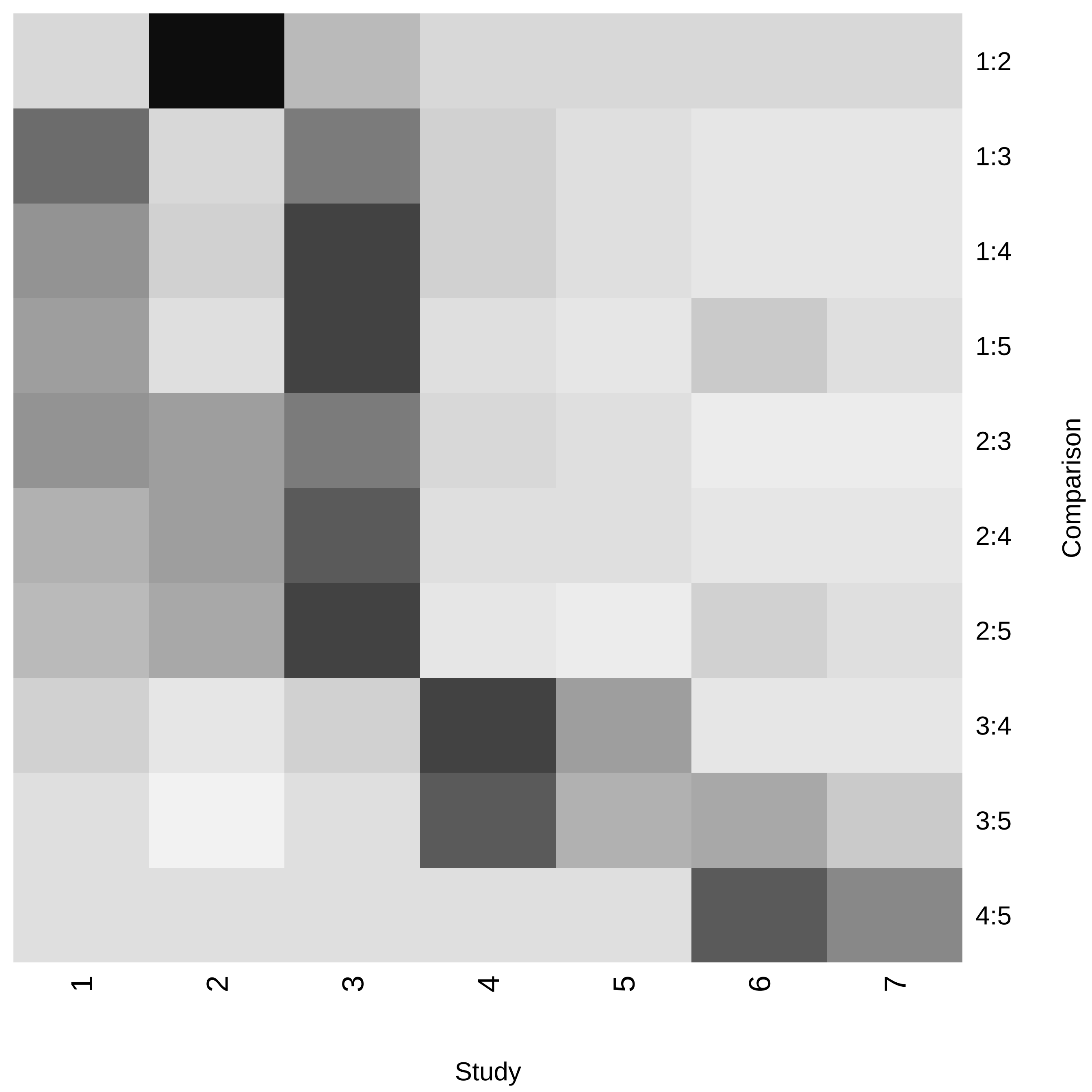
1:2
1:3
1:4
1:5
2:3
2:4
2:5
3:4
3:5
4:5
Comparison
1
2
3
4
5
6
7
Study

Full network

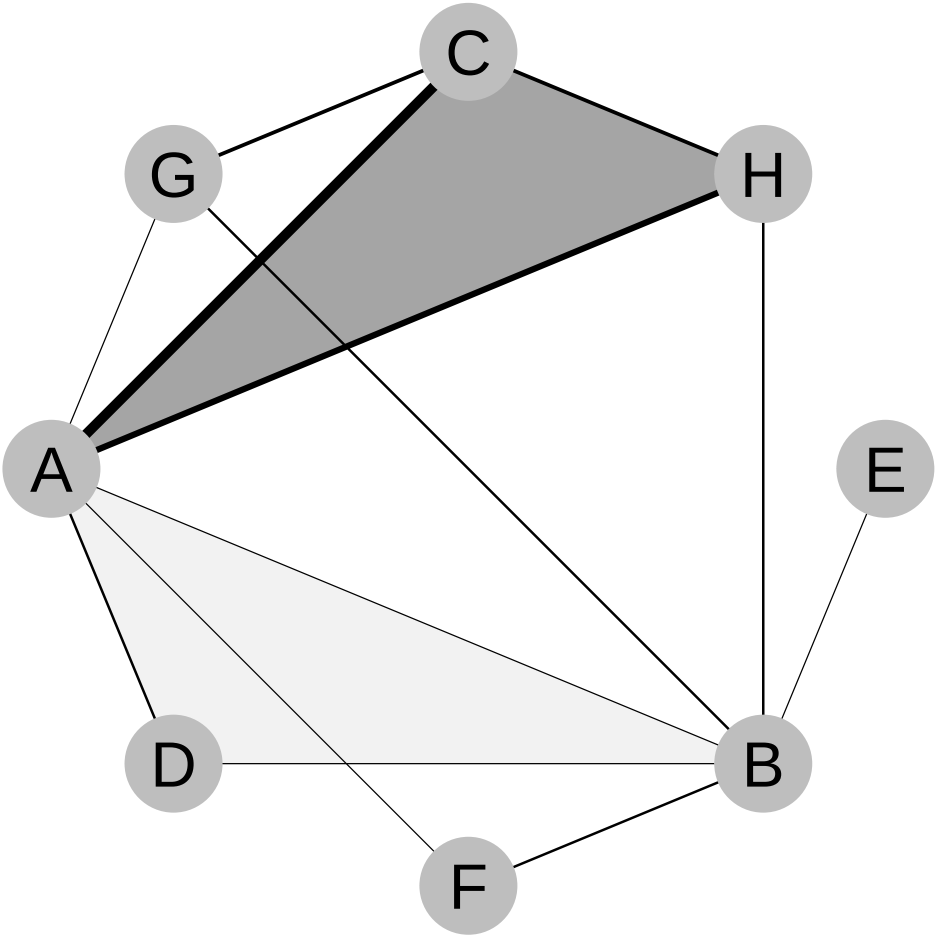

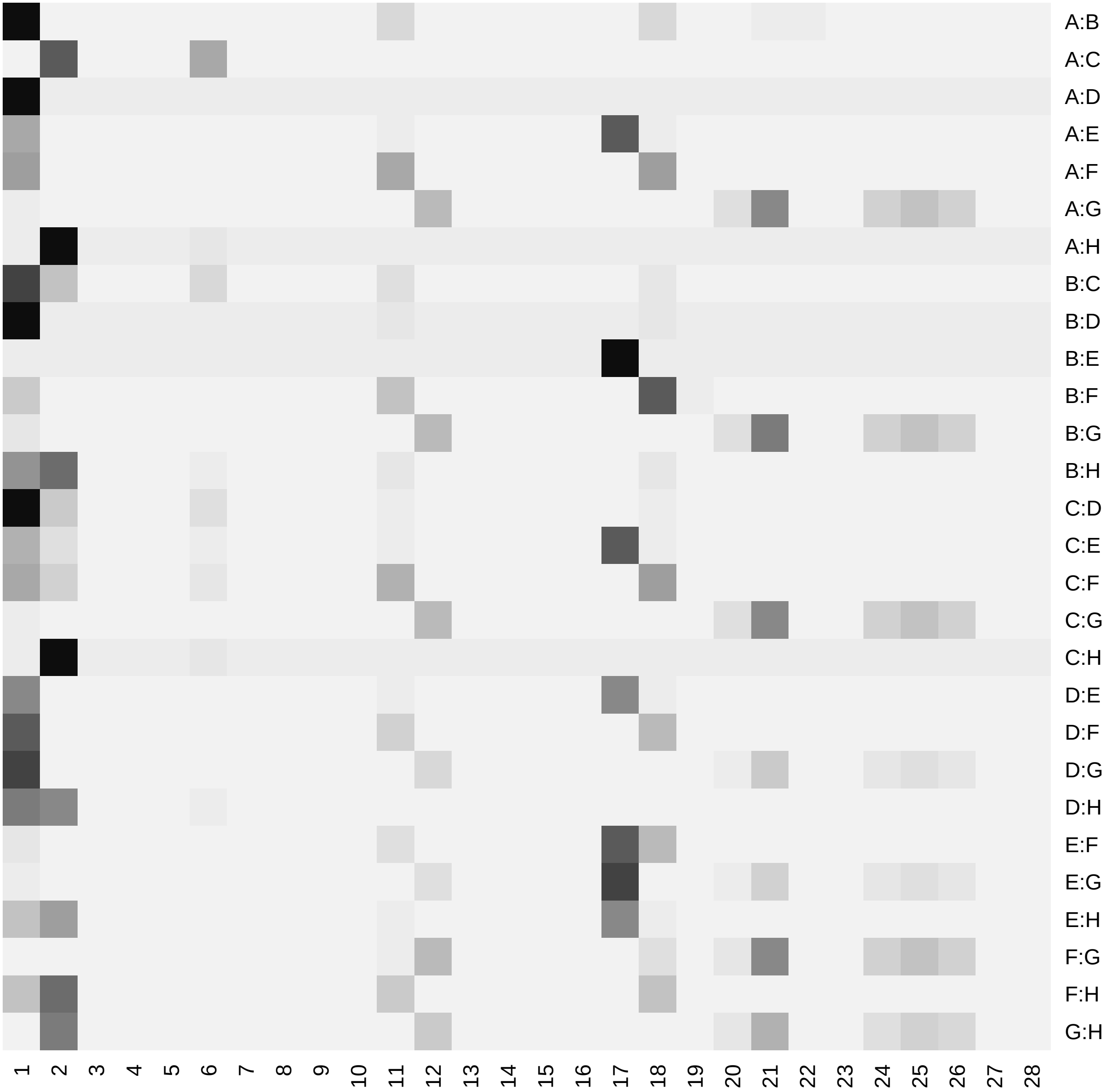
A:B
A:C
A:D
A:E
A:F
A:G
A:H
B:C
B:D
B:E
B:F
B:G
B:H
C:D
C:E
C:F
C:G
C:H
D:E
D:F
D:G
D:H
E:F
E:G
E:H
F:G
F:H
G:H
1
2
3
4
5
6
7
8
9
10
11
12
13
14
15
16
17
18
19
20
21
22
23
24
25
26
27
28

Comparison: other vs 'A'
(Fixed Effect Model)

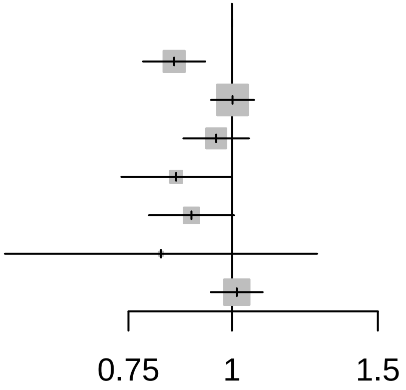

Treatment	OR	95%−CI
streptokinase	1.00	
accelerated alteplase	0.85	[0.78; 0.93]
alteplase	1.00	[0.94; 1.06]
streptokinase + alteplase	0.96	[0.87; 1.05]
tenecteplase	0.86	[0.74; 1.00]
reteplase	0.89	[0.79; 1.01]
urokinase	0.82	[0.53; 1.27]
anistreptilase	1.01	[0.94; 1.09]

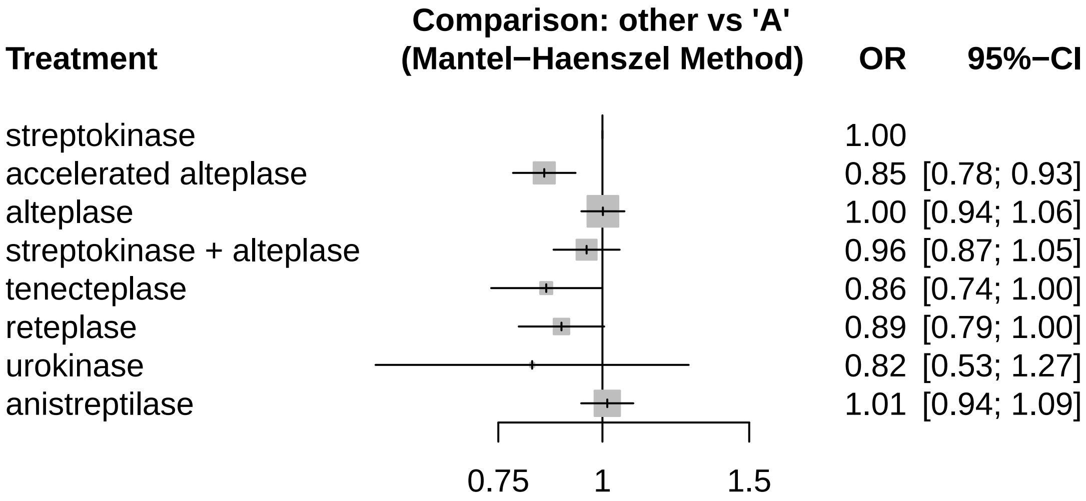
Comparison: other vs 'A'
(Mantel−Haenszel Method)
Treatment
OR
95%−CI
streptokinase 1.00
accelerated alteplase 0.85 [0.78; 0.93]
alteplase 1.00 [0.94; 1.06]
streptokinase + alteplase 0.96 [0.87; 1.05]
tenecteplase 0.86 [0.74; 1.00]
reteplase 0.89 [0.79; 1.00]
urokinase 0.82 [0.53; 1.27]
anistreptilase 1.01 [0.94; 1.09]
0.75
1
1.5
